# Preclinical effects of naringin on bone outcomes in animal models of osteoporosis: a systematic review and meta-analysis

**DOI:** 10.3389/fphar.2026.1945663

**Published:** 2026-09-18

**Authors:** Chang Tan, Xiaonian Zhou, Yanming Xie, Yingjie Zhi

**Affiliations:** Institute of Basic Research in Clinical Medicine, China Academy of Chinese Medical Sciences, Beijing, China

**Keywords:** animal models, bone microarchitecture, bone mineral density, bone turnover markers, naringin, osteoporosis

## Abstract

**Background:**

Naringin, a flavanone glycoside derived from citrus plants and other botanical sources, has shown potential skeletal protective effects in preclinical studies. However, quantitative evidence regarding its effects across different osteoporosis models and skeletal outcomes remains limited.

**Methods:**

A systematic review and meta-analysis of controlled preclinical animal studies evaluating naringin in osteoporosis-related bone-loss models was conducted. Five databases were searched from January 2010 to May 2026. Standardised mean differences (SMDs) expressed as Hedges’ *g*, were pooled using random-effects models. Twenty-six reports representing 24 independent experimental cohorts were included. Certainty of evidence was assessed using an adapted Grading of Recommendations Assessment, Development and Evaluation (GRADE) framework for preclinical animal studies.

**Results:**

Naringin was associated with higher bone mineral density (BMD; (SMD = 2.30, 95% CI 1.88–2.72), bone volume fraction (BV/TV; SMD = 3.36, 95% CI 2.17-4.55), trabecular thickness (Tb.Th; SMD = 2.35, 95% CI 1.49-3.21), trabecular number (Tb.N; SMD = 3.30, 95% CI 1.98-4.61), and lower trabecular separation (Tb.Sp; SMD = -2.92, 95% CI -4.01 to -1.83). Favourable pooled effects were also observed for several bone turnover markers. Prediction intervals excluded the null for BMD and C-terminal telopeptide of type I collagen (CTX-1) but crossed the null for most other outcomes. Sensitivity analyses generally preserved the direction of the pooled effects, although potential small-study effects were detected for several structural outcomes. Dose-response associations varied across analytical specifications and did not establish a stable optimal dose. Certainty of evidence was low for CTX-1 and very low for all other outcomes.

**Conclusion:**

Naringin showed a broad favourable preclinical signal for improving bone density, trabecular microarchitecture, and selected bone turnover markers in animal models of osteoporosis. The evidence was comparatively more robust for BMD and CTX-1, whereas heterogeneity, methodological limitations, and low certainty of evidence reduced confidence in the magnitude and reproducibility of effects across experimental settings. These findings support further investigation of naringin as a potential skeletal protective agent, with well-designed preclinical studies needed to clarify reproducibility, dose‐response relationships, and translational relevance.

**Systematic Review Registration:**

identifier CRD420261419299.

## 1 Introduction

Osteoporosis is a systemic skeletal disorder characterised by reduced bone mineral density (BMD), deteriorated bone microarchitecture, and increased fragility-fracture risk (Samelson et al., 2019). It affects approximately 200 million people worldwide (Shen et al., 2022) and causes substantial morbidity, mortality, and healthcare expenditure, particularly among postmenopausal women and older adults (Liang et al., 2025). Its pathophysiology centres on an imbalance between osteoblast-mediated bone formation and osteoclast-mediated resorption, driven by factors including oestrogen deficiency, glucocorticoid excess, mechanical disuse, metabolic disease, and cellular senescence (Rolvien and Amling, 2022; Cheng et al., 2022).

Current treatments include anti-resorptive agents, such as bisphosphonates and denosumab, and anabolic agents, including teriparatide and romosozumab (Reid and Billington, 2022). Despite reducing fracture risk, these therapies have important limitations. Bisphosphonates are associated with osteonecrosis of the jaw, atypical subtrochanteric fractures, atypical femoral fractures, and gastrointestinal intolerance, while denosumab discontinuation can cause rebound bone loss (Meyyur Aravamudan and Er, 2019; Anastasilakis et al., 2021). Anabolic therapies are costly and have restricted treatment durations (Eastell et al., 2019). These limitations have encouraged continued investigation of naturally derived compounds with potential effects on bone remodelling.

Plant-rich dietary patterns, including the Mediterranean diet, provide abundant polyphenols and flavonoids that may influence bone homeostasis through oxidative stress, inflammation, and osteoblast‐osteoclast coupling. Citrus-derived flavanones are of particular interest because of their antioxidant and anti-inflammatory properties and potential roles in age-related diseases (Perrone et al., 2025). However, the effects of individual flavonoids cannot be inferred from dietary patterns alone and require metabolite-specific evaluation.

Naringin (4′,5,7-trihydroxyflavanone-7-rhamnoglucoside; CAS 10236‐47-2) is a flavanone glycoside found primarily in citrus plants, particularly grapefruit, commonly referred to as Citrus × paradisi Macfad. (Rutaceae; treated by POWO as a synonym of Citrus × aurantium f. aurantium). This review evaluated naringin as an isolated or purified plant-derived metabolite rather than a botanical extract (Stabrauskiene et al., 2022). Naringin is structurally distinct from its aglycone naringenin, and its rhamnosylglucose moiety confers different pharmacokinetic and pharmacodynamic properties (Xu et al., 2020). Mechanistic studies suggest that naringin promotes osteogenic differentiation through Wnt/β-catenin signalling, including GSK-3β inhibition, β-catenin stabilisation, and RUNX2 upregulation (Cui et al., 2025). It may also regulate the OPG/RANKL axis (Ang et al., 2011) and modulate the PI3K/Akt/mTOR, JAK2/STAT3, and NF-κB pathways (Ge and Zhou, 2021; Wang et al., 2022).

Despite increasing mechanistic evidence, quantitative synthesis remains limited. A previous review included a relatively small evidence base and focused mainly on ovariectomised rodents and BMD-related outcomes (Zhu et al., 2021), leaving trabecular microarchitecture and bone turnover markers insufficiently characterised. Since 2020, the evidence has expanded to include glucocorticoid- and disuse-induced osteoporosis, diabetes-associated bone loss, metabolic syndrome models, and a broader range of skeletal outcomes (Gan et al., 2023), warranting an updated synthesis.

We therefore conducted a prospectively registered systematic review and meta-analysis (PROSPERO: CRD420261419299) of animal studies identified from five databases, including Chinese-language literature. Primary outcomes were BMD and BV/TV, with subgroup analyses exploring heterogeneity by species and osteoporosis model. Secondary analyses examined Tb.Th, Tb.N, Tb. Sp, five bone turnover markers, funnel-plot asymmetry, and study-level dose associations.

## 2 Methods

### 2.1 Protocol registration

This systematic review and meta-analysis was conducted and reported in accordance with the Preferred Reporting Items for Systematic Reviews and Meta-Analyses (PRISMA) 2020 statement. The review protocol was registered with the International Prospective Register of Systematic Reviews (PROSPERO; registration number: CRD420261419299) before completion of data extraction, risk-of-bias assessment, and quantitative synthesis.

### 2.2 Search strategy

A systematic literature search was independently conducted by two reviewers across five electronic databases: PubMed, Web of Science, Embase, China National Knowledge Infrastructure (CNKI), and Wanfang Data. The search covered publications in English or Chinese from January 2010 to May 2026; a preliminary scoping search did not identify any eligible controlled animal studies published before 2010 (Supplementary 1, Table S1). The search strategy combined Medical Subject Headings (MeSH) terms and free-text keywords across three concept groups: (A) intervention terms for naringin, including its established chemical names and spelling variants, with naringenin and other structurally related but distinct flavonoids explicitly excluded; (B) condition (osteoporosis and related bone-loss terms), and (C) animal models (species and induction methods), linked by Boolean AND/OR operators (Supplementary 1, Table S2-S3). The complete search strings for all five databases are provided in the Supplementary Materials. Reference lists of all included studies were manually screened for additional eligible records. Discrepancies between reviewers were resolved by discussion with a third reviewer.

### 2.3 Inclusion and exclusion criteria

Inclusion criteria: (1) controlled animal experiments published as peer-reviewed journal articles in English or Chinese; (2) validated animal models of osteoporosis of any species, including ovariectomy (OVX)-induced, glucocorticoid-induced, disuse-induced, diabetic, and metabolic models; (3) naringin administered as the primary intervention, with no restriction on dose, route, or duration; (4) at least one control group (blank, model, sham-operated, or positive drug control); and (5) at least one prespecified outcome measure reported.

Exclusion criteria: (1) *in vitro* or *ex vivo* studies, reviews, meta-analyses, case reports, editorials, or conference abstracts; (2) studies with unavailable full texts, duplicate publications, or incomplete or non-extractable data; (3) studies in which naringin was administered only as part of a fixed combination with other active compounds without a naringin-only arm; (4) studies using fracture-healing or angiogenesis models, regardless of whether bone density or microstructural outcomes were reported.

### 2.4 Study selection

Study selection followed three sequential stages under PRISMA 2020 guidelines. All retrieved records were first imported into Zotero and deduplicated automatically. Two independent reviewers then screened titles and abstracts to exclude clearly ineligible studies. Full texts of potentially eligible studies were subsequently assessed against the prespecified criteria. Disagreements at each stage were resolved by discussion or arbitration by a third reviewer. The selection process and reasons for full-text exclusion are documented in the PRISMA 2020 flow diagram (Figure 1).

**FIGURE 1:**
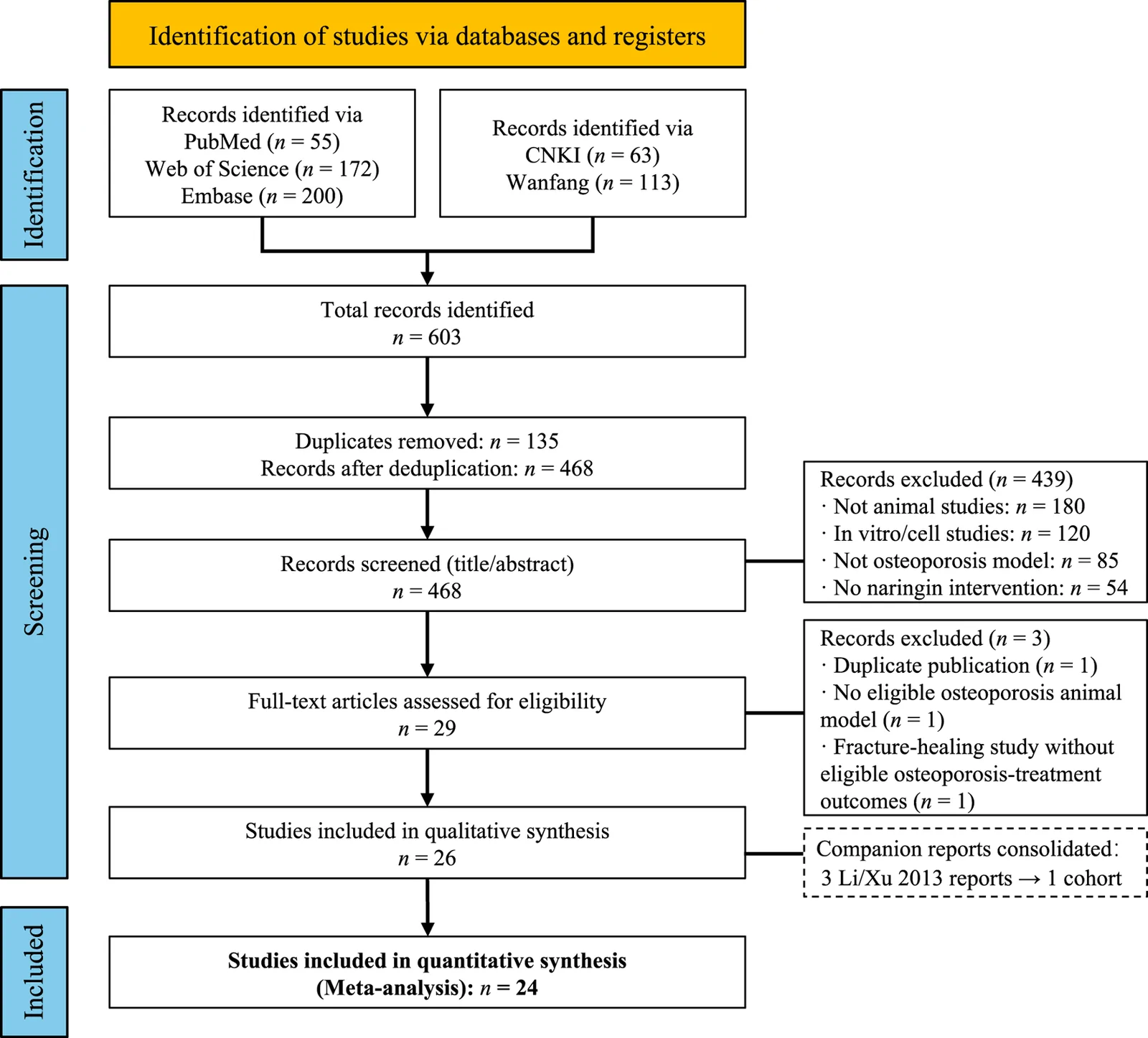
PRISMA 2020 flow diagram of the study selection process.

### 2.5 Outcome measures

The primary outcomes were BMD and bone volume fraction (BV/TV), reflecting overall bone quantity and trabecular bone volume, respectively. Secondary outcomes specified in the registered protocol included three additional trabecular microstructural parameters, namely, trabecular thickness (Tb.Th), trabecular number (Tb.N), and trabecular separation (Tb.Sp) (Bouxsein et al., 2010), together with osteocalcin (OCN) and C-terminal telopeptide of type I collagen (CTX-1). Procollagen type I N-terminal propeptide (P1NP), tartrate-resistant acid phosphatase (TRAP), and alkaline phosphatase (ALP) were added during the review as additional exploratory bone turnover outcomes because they were reported in multiple eligible studies (Bhattoa et al., 2021; Brown et al., 2022).

### 2.6 Data extraction

A standardised spreadsheet was used to extract publication details, animal characteristics (species, strain, sex, age, group size, model type, and induction method), intervention protocols (naringin dose, route, duration, and control type), and prespecified outcomes reported as mean ± standard deviation. Two reviewers independently extracted numerical data from figures using WebPlotDigitizer (version 5.2). To obtain one effect size per study and outcome, the distal femur was prioritised when multiple skeletal sites were reported; otherwise, the most prominently reported site was selected. The final time point, micro-CT rather than DXA for the same site, and the left side for bilateral measurements were used.

Reports describing the same cohort and experimental protocol were consolidated to prevent double-counting. Three reports by Li and Xu published in 2013 involved the same cohort of 24 ovariectomised rats, as indicated by identical group sizes, naringin doses, control treatments, and intervention durations, and were treated as one independent cohort. BV/TV, Tb.Th, and Tb. Sp were obtained from the reports by Li et al. The cohort did not contribute to the BMD analysis because only relative changes, without extractable group-level means and standard deviations, were reported. The companion report by Xu et al. provided femoral biomechanical strength and tibial trabecular area data, which confirmed the cohort overlap but were not prespecified meta-analytic outcomes. When outcomes overlapped across reports, values were cross-checked and the most complete extractable dataset was used; each cohort contributed only once per outcome.

For multi-dose studies with a shared control, one arm was selected using a prespecified dose-position rule: the middle-tested dose for three-dose studies and the lower tested dose for two-dose studies (Axon et al., 2023). Selection was independent of statistical significance or reported efficacy, and all multi-dose studies were rechecked against this rule. This approach avoided repeated use of shared controls in the primary analysis but excluded the remaining dose arms. Remaining dose arms were excluded only from the primary effect-size synthesis but were retained for dose-response analyses.

Meta-analytic comparisons used untreated or vehicle-treated osteoporotic model animals as controls; blank, sham-operated, and positive-drug groups were not used as reference groups. Disagreements were resolved through discussion or consultation with a third reviewer.

The reported source or supplier, catalogue or batch details, purity, and analytical quality control of naringin were extracted. Botanical origin was recorded only when isolation or extraction from plant material was explicitly reported and was not inferred for commercially purchased naringin. Reported plant names were verified using Plants of the World Online (POWO).

### 2.7 Risk of bias assessment

The methodological quality of each included study was independently assessed by two reviewers using the SYRCLE Risk of Bias Tool for Animal Studies, which evaluates bias across 10 domains: sequence generation, baseline characteristics comparability, allocation concealment, random housing, blinding of caregivers and investigators, random outcome assessment, blinding of outcome assessors, incomplete outcome data, selective outcome reporting, and other potential bias sources. Each domain was rated low risk, unclear risk, or high risk. Discrepancies were resolved by consensus or arbitration by a third reviewer.

### 2.8 Statistical analysis

All analyses were performed in R (version 4.5.1) using the *meta* and *metafor* packages, with two-tailed *p* < 0.05 indicating statistical significance. Continuous outcomes were expressed as SMDs, calculated as Hedges’ *g* with small-sample correction, and reported with 95% CIs. Random-effects models used REML estimation and Knapp–Hartung adjustment. Heterogeneity was assessed using Cochran’s Q, *I* ^2^, and *τ* ^2^; *I* ^2^ was interpreted as low (<25%), moderate (25%–50%), substantial (50%–75%), or considerable (>75%). Prediction intervals were calculated for outcomes with at least five studies.

Animal species (rat vs. mouse) (Riley et al., 2011) and osteoporosis model type (OVX, disuse, or other) were prespecified as potential sources of heterogeneity. Species subgroup analyses were conducted for BMD and BV/TV, and exploratory model-type analyses for outcomes with at least five studies and sufficient representation across model categories (BMD, BV/TV, Tb.Th, Tb.N, Tb. Sp, and OCN). Categories represented by one study were described narratively. Subgroup differences were tested using chi-squared interaction tests.

Primary analyses included one prespecified representative dose arm per study and outcome. Dose–response analyses retained all eligible arms and accounted for dependence from shared controls through the sampling variance‐covariance matrix and multilevel random-effects models with study-cluster robust inference. Dose was modelled on the raw and log10 scales, with meta-regression restricted to outcomes with at least 10 eligible independent studies. Three-dimensional plots descriptively displayed dose, duration, and study identity for the five structural outcomes with at least 10 studies; they were not used for dose‐response inference.

For outcomes with at least 10 studies, small-study effects were assessed using funnel plots and Egger’s test. Because SMD is mathematically associated with its standard error, asymmetry was interpreted as potential small-study effects rather than definitive publication bias. Post-hoc trim-and-fill analyses using the L0 estimator were applied to the representative-dose dataset, with estimates treated as sensitivity results rather than corrected effects because of their dependence on assumptions about asymmetry and heterogeneity.

Leave-one-out analyses were performed for outcomes with at least five studies. Additional *post hoc* analyses for BV/TV and Tb.N excluded visually identified extreme effects (|SMD| > 8), although all studies remained in the primary analyses. DerSimonian–Laird models were fitted for BMD and BV/TV to assess sensitivity to *τ* ^2^ estimation.

To evaluate dose-arm selection, analyses were repeated using the highest available arm in each multi-dose study while retaining one estimate per study and outcome. The same REML, Knapp‐Hartung, and prediction-interval procedures were applied. Highest-dose study-level meta-regressions were conducted for the seven outcomes with at least 10 studies, and their results were compared with the representative-dose and all-dose-arm multilevel analyses.

Multivariable meta-regression was considered when at least 10 independent study clusters were available per moderator coefficient and each categorical subgroup contained at least five clusters. Candidate models combined raw or log10 dose with species or model type. Influence diagnostics for the all-dose-arm models included dose-arm-level externally studentised residuals, study-cluster Cook’s distances, and diagonal hat values. Models were also refitted after excluding doses ≥300 mg/kg/day or the outcome-specific maximum dose. Potential influence was defined as |studentised residual| > 2, Cook’s distance >4/k, or hat value > 2p/n, where k, p, and n denote study clusters, model parameters, and dose-arm comparisons, respectively. These *post hoc* thresholds were not used as automatic exclusion criteria.

Subgroup, meta-regression, funnel-plot, influence, and *post hoc* sensitivity analyses were exploratory. No multiplicity adjustment was applied, and findings were interpreted as hypothesis-generating rather than confirmatory (Menyhárt and Győrffy, 2025).

### 2.9 Certainty of evidence

Certainty for each pooled outcome was assessed using an adapted GRADE framework for preclinical animal intervention studies (Hooijmans et al., 2018). Five predefined domains were considered: risk of bias, inconsistency, imprecision, indirectness, and publication bias or small-study effects. Evidence from controlled *in vivo* experiments initially started at high certainty and was downgraded by one level for serious concerns or two levels for very serious concerns, yielding high, moderate, low, or very-low certainty.

Risk of bias was determined from outcome-relevant SYRCLE judgements, with downgrading when most evidence came from studies at unclear or high risk in key domains, including randomisation, allocation concealment, random housing, blinding, and selective reporting. Inconsistency was judged from the direction and dispersion of estimates, confidence-interval overlap, *I* ^2^, *τ* ^2^, subgroup findings, and prediction intervals rather than *I* ^2^ alone. Serious inconsistency required substantial unexplained heterogeneity with materially dispersed estimates or a prediction interval crossing the null; very serious inconsistency required considerable heterogeneity with widely dispersed or conflicting estimates. Judgements were anchored to the primary representative-dose analysis, with highest-dose, all-dose-arm, influence, and small-study-effect analyses used as supporting evidence.

Imprecision was evaluated from the amount of information, confidence-interval width, and whether the interval encompassed materially different conclusions, including no effect or effects in the opposite direction. As validated minimally important differences were unavailable, judgements were based on the range of compatible effects rather than statistical significance alone. Indirectness was considered in relation to both the preclinical question and human translation (Bahadoran et al., 2020). Evidence limited to rodent models, surrogate outcomes, or doses without established human exposure equivalence was considered seriously indirect, with very serious indirectness assigned when correspondence with the intended clinical context was particularly limited.

Publication bias or small-study effects were evaluated using search comprehensiveness, study number and size, funnel plots, Egger’s test, and trim-and-fill analyses when at least 10 studies were available. Downgrading required evidence suggesting that the pooled effect might be exaggerated; lack of formal asymmetry testing for outcomes with fewer than 10 studies was not itself considered evidence of bias. Potential upgrading factors included a large effect, a consistent dose–response gradient, or plausible residual confounding that would reduce the observed effect. A large SMD alone did not justify upgrading, and dose–response evidence was considered credible only when consistent across models and robust to influence and high-dose sensitivity analyses. Two reviewers independently rated each outcome, resolving disagreements through discussion or consultation with a third reviewer.

## 3 Results

### 3.1 Literature selection

A comprehensive search of five databases identified 603 records in total (PubMed: 55; Web of Science: 172; Embase: 200; CNKI: 63; Wanfang: 113). After removing 135 duplicates, 468 records remained for title and abstract screening, of which 439 were excluded (not animal studies, *n* = 180; in vitro or cell studies, *n* = 120; not osteoporosis-related models, *n* = 85; no naringin intervention, *n* = 54). The remaining 29 records underwent full-text assessment. Three records were excluded at this stage: one report was excluded because it represented an overlapping publication without additional eligible data, one did not use an eligible osteoporosis or bone-loss animal model, and one evaluated fracture healing rather than the treatment of osteoporosis (Appendix A). Thus, 26 eligible reports were included in the qualitative synthesis. Three reports published by Li and Xu in 2013 described the same animal cohort and experimental protocol and were therefore consolidated into a single study entry. Consequently, the 26 eligible reports represented 24 independent experimental cohorts for quantitative meta-analysis. The PRISMA 2020 flow diagram is presented in Figure 1.

### 3.2 Characteristics of included studies

The 24 included experimental cohorts were published between 2010 and 2025. Nineteen used rats (predominantly Sprague-Dawley) and five used mice (predominantly C57BL/6). Sixteen used female animals and eight used male animals. Animal age at the time of osteoporosis induction ranged from 1 to 8 months. The most common model was ovariectomy (OVX; *n* = 14), followed by disuse-induced models including hindlimb suspension and sciatic neurectomy (*n* = 4), streptozotocin-induced diabetic bone loss (*n* = 4), glucocorticoid-induced models (*n* = 2), retinoic acid-induced (*n* = 1), and high-fructose diet-induced metabolic syndrome (*n* = 1). Reported naringin doses ranged from 4 to 1,500 mg/kg/day; most cohorts used oral gavage (*n* = 20) and four used subcutaneous injection. Treatment durations ranged from 7 days to 3 months. Detailed characteristics of animal models and intervention protocols are provided in Tables 1, 2.

**TABLE 1:** Characteristics of animal models and species in the included experimental cohorts.

Study (first author, year)	Year	Species/Strain/Sex	Age (months)	n/group	Sex	Osteoporosis model type	Induction method	Subgroup
(Shi et al., 2023)	2023	Mouse, C57BL/6J	6	15	Male	Disuse (tail suspension)	30-day tail suspension	Disuse
(Li et al., 2012)	2012	Rat, SD	4	10	Female	OVX	Bilateral ovariectomy	OVX
(Rivoira et al., 2022)	2022	Rat, Wistar	2	9	Male	Metabolic syndrome (FRD)	10% fructose drinking water for 30 d	Metabolic syndrome
(Lyu et al., 2016)	2016	Rat, SD	8	18	Male	Disuse (tail suspension)	Hindlimb suspension (HLS), 8 weeks	Disuse
(Shangguan et al., 2022)	2022	Rat, SD	6	12	Female	OVX	Bilateral OVX, intervention 3 months post-OVX	OVX
(Lan et al., 2025)	2025	Rat, SD	2	10	Female	OVX	Bilateral OVX; intervention 8 weeks post-OVX	OVX
(Zhang et al., 2025)	2025	Rat, SD	8	10	Female	OVX	Bilateral OVX; intervention 1 week post-OVX for 12 weeks	OVX
(Shangguan et al., 2017)	2017	Rat, SD	6	12	Female	OVX	Bilateral OVX; intervention from day 3 post-OVX for 3 months	OVX
(Pang et al., 2010)	2010	Mouse, C57BL/6J	1	7–8	Female	OVX	Bilateral OVX; 2-week recovery, then intervention for 6 weeks	OVX
(Li et al., 2018)	2018	Rat, Wistar	1	8	Male	GIOP (IBD + DEX)	DSS-induced IBD + dexamethasone 1 mg/kg/d i.p. for 5 weeks	GIOP
(Sun et al., 2014)	2014	Rat, SD	2	10	Female	OVX	Bilateral OVX; intervention 2 months post-OVX for 60 d	OVX
(Lv et al., 2015)	2015	Rat, SD	6	6	Male	Disuse (sciatic neurectomy)	Right sciatic nerve transection (USN); treatment from 4 weeks pre-surgery for 8 weeks	Disuse
(Ma et al., 2016)	2016	Rat, SD	6	15	Male	Disuse (sciatic neurectomy)	Right sciatic nerve transection 5 mm (USN); treatment from 4 weeks pre-surgery for 8 weeks	Disuse
(Li et al., 2013)	2013	Rat, SD	3	6	Female	OVX	Bilateral OVX; 3-month modelling period, then intervention for 2 months	OVX
(Rivoira et al., 2018)	2018	Rat, Wistar	2	8	Male	STZ-induced diabetic bone loss	STZ 60 mg/kg i.p.; intervention from day 3 post-STZ for 30 days	Diabetic
(Ge and Zhou, 2021)	2021	Rat, SD	1	12	Female	GIOP (DEX)	DEX sodium phosphate 1 mg/kg i.m. twice weekly for 8 weeks	GIOP
(Li et al., 2025)	2025	Mouse, C57BL/6	8	7	Female	OVX	Bilateral OVX; 4-week recovery, then intervention for 8 weeks	OVX
(Cui et al., 2025)	2025	Mouse, C57BL/6	8	6	Female	OVX	Bilateral OVX; intervention 1 week post-OVX for 12 weeks	OVX
(Li et al., 2014)	2014	Rat, SD	6	5	Female	OVX	Bilateral OVX; intervention 60 days post-OVX for 60 days	OVX
(Sun et al., 2015)	2015	Rat, SD	3	15	Female	OVX	Bilateral OVX; 3-month modelling, then 300 mg/kg for 60 days	OVX
(Jin et al., 2022)	2022	Rat, SD	8	3	Female	Retinoic acid (RA)-induced	RA 90 mg/kg oral gavage for 14 days; naringin for 30 days post-modelling	Other (RA)
(Wang et al., 2015)	2015	Mouse (strain NR)	6	8	Female	OVX	OVX at 1 month of age; 2-week recovery, then intervention for 6 weeks	OVX
(Rodríguez et al., 2024)	2024	Rat, Wistar	NR	6	Male	STZ-induced diabetic bone loss	STZ i.p.; intervention for 30 days	Diabetic
(Wang et al., 2022)	2022	Rat, SD	8	10	Female	OVX	Bilateral OVX; intervention 60 days post-OVX for 60 days	OVX

**TABLE 2:** Intervention protocols and naringin dosing characteristics of the included experimental cohorts.

Study (first author, year)	Year	Naringin dose used in meta-analysis (mg/kg/day)	Route of administration	Treatment duration	Control group(s)	Notes on dose selection
(Shi et al., 2023)	2023	40 (middle dose of 3-arm study: 20/40/80)	Subcutaneous injection	30 days	Tail-suspension model (TS) control	Three naringin dose groups; middle dose (40 mg/kg) selected according to the prespecified dose-position rule
(Li et al., 2012)	2012	4	Oral gavage	13 weeks	Sham-operated + OVX model	Single-dose study; lowest reported dose in the evidence base
(Rivoira et al., 2022)	2022	40	Subcutaneous injection	27 days	Normal diet control + FRD model group	Single-dose study
(Lyu et al., 2016)	2016	100 (middle dose of 3-arm study: 30/100/300)	Oral gavage	8 weeks	Normal control + HLS model group	Three naringin dose groups; middle dose (100 mg/kg) selected according to the prespecified dose-position rule
(Shangguan et al., 2022)	2022	200	Oral gavage	3 months	Sham-operated + OVX model	Single-dose study
(Lan et al., 2025)	2025	100 (lower of two doses: 100/400)	Oral gavage	7 days	Sham-operated + OVX model	Two naringin dose groups; lower dose selected according to the prespecified dose-position rule
(Zhang et al., 2025)	2025	100	Oral gavage	12 weeks	Sham-operated + OVX model	Single-dose study
(Shangguan et al., 2017)	2017	100 (lower of two doses: 100/200)	Oral gavage	3 months	Sham-operated + OVX model	Two naringin dose groups; lower dose selected according to the prespecified dose-position rule
(Pang et al., 2010)	2010	200 (lower of two doses: 200/400)	Oral gavage	6 weeks	Sham-operated + OVX model	Two naringin dose groups; lower dose (200 mg/kg) selected according to the prespecified dose-position rule
(Li et al., 2018)	2018	100 (lower of two doses: 100/200)	Oral gavage	5 weeks	Normal control + IBD + DEX model	Two naringin dose groups; lower dose selected according to the prespecified dose-position rule
(Sun et al., 2014)	2014	100 (middle dose of 3-arm study: 40/100/200)	Oral gavage	60 days	Sham-operated + OVX model	Three naringin dose groups; middle dose (100 mg/kg) selected according to the prespecified dose-position rule
(Lv et al., 2015)	2015	100 (middle dose of 3-arm study: 30/100/300)	Oral gavage	8 weeks	Sham-operated + USN model	Three naringin dose groups; middle dose (100 mg/kg) selected according to the prespecified dose-position rule
(Ma et al., 2016)	2016	100 (middle dose of 3-arm study: 30/100/300)	Oral gavage	8 weeks	Sham-operated + USN model	Three naringin dose groups; middle dose (100 mg/kg) selected according to the prespecified dose-position rule; companion paper to Lv 2015
(Li et al., 2013)	2013	300 (middle of 3-arm study: 60/300/1,500)	Oral gavage	2 months	Sham-operated + PBS-treated OVX	Three naringin dose groups; middle dose (300 mg/kg) selected according to the prespecified dose-position rule
(Rivoira et al., 2018)	2018	40 (lower of two doses: 40/80; per prespecified rule)	Subcutaneous injection	30 days	Normal control + STZ model	Two naringin dose groups; lower dose selected according to the prespecified dose-position rule
(Ge and Zhou, 2021)	2021	200	Oral gavage	8 weeks	Normal PBS control + GIOP model	Single-dose study
(Li et al., 2025)	2025	20 (middle of 3-arm study: 10/20/40)	Oral gavage	8 weeks	Sham-operated + OVX model	Three naringin dose groups; middle dose (20 mg/kg) selected according to the prespecified dose-position rule
(Cui et al., 2025)	2025	15 (lower of two doses: 15/30)	Oral gavage	12 weeks	Sham-operated + OVX model	Two naringin dose groups; lower dose selected according to the prespecified dose-position rule
(Li et al., 2014)	2014	100 (middle of 3-arm study: 40/100/200)	Oral gavage	60 days	Sham-operated + OVX model	Three naringin dose groups; middle dose (100 mg/kg) selected according to the prespecified dose-position rule
(Sun et al., 2015)	2015	300	Oral gavage	60 days	Sham-operated + OVX model	Single-dose study; separate study from sun XL 2014
(Jin et al., 2022)	2022	50	Oral gavage	30 days	Normal control + RA model	Single-dose study; RA model (retinoic acid)
(Wang et al., 2015)	2015	5	Oral gavage	6 weeks	Sham-operated + OVX model	Single-dose study; low dose relative to other included studies
(Rodríguez et al., 2024)	2024	40	Subcutaneous injection	30 days	Normal control + STZ model	Single-dose study
(Wang et al., 2022)	2022	300	Oral gavage	60 days	Sham-operated + OVX model	Single-dose study

Naringin was treated as an isolated or purified plant-derived metabolite rather than a botanical extract. Among the 24 included cohorts, information on the source or supplier of naringin was reported in 19 cohorts (79%), whereas purity was reported in only seven cohorts (29%) and analytical quality-control methods, such as HPLC identification, were reported in only two cohorts (8%) (Table 3). Most studies used commercially purchased or institution-supplied naringin, for which the original botanical source was not reported and could not be independently inferred. Only one study explicitly reported that naringin was self-extracted from plant material. The incomplete reporting of supplier details, purity, and analytical quality-control procedures represents an important limitation in intervention reproducibility and may contribute to between-study heterogeneity.

**TABLE 3:** Source or supplier, purity, and analytical quality-control reporting for naringin used in the included experimental cohorts.

Study	Source or supplier	Purity (%)	Quality control reported
Shi et al., 2023	Not reported	Not reported	Not reported
Li et al., 2012	School of pharmaceutical sciences, central south university, changsha, China	>98	Not reported
Rivoira et al., 2022	Sigma-aldrich, St. Louis, MO, United States of America	Not reported	Not reported
Lyu et al., 2016	Sigma, United States of America	Not reported	Not reported
Shangguan et al., 2022	Sigma-aldrich	≥99	Not reported
Lan et al., 2025	Sigma-aldrich, United States of America	≥99.99	Not reported
Zhang et al., 2025	Chengdu must bio-technology co., Ltd., chengdu, China	Not reported	Not reported
Shangguan et al., 2017	Sigma-aldrich, St. Louis, MO, United States of America	Not reported	Not reported
Pang et al., 2010	Sigma-aldrich, St. Louis, MO, United States of America	Not reported	Not reported
Li et al., 2018	Not reported	Not reported	Not reported
Sun et al., 2014	Xi’an guanyu biotechnology co., Ltd., Xi’an, China	98	Not reported
Lv et al., 2015	Not reported	Not reported	Not reported
Ma et al., 2016	Sigma-aldrich, St. Louis, MO, United States of America	Not reported	Not reported
Li et al., 2013	Sigma-aldrich, St. Louis, MO, United States of America; Cat# 71162, Lot# BCBD7489V	>95	HPLC
Rivoira et al., 2018	Sigma-aldrich, St. Louis, MO, United States of America	Not reported	Not reported
Ge and Zhou 2021	Selleck chemicals, United States of America	Not reported	Not reported
Li et al., 2025	MedChemExpress (MCE), United States of America	Not reported	Not reported
Cui et al., 2025	MedChemExpress, United States of America	99.75	Not reported
Li et al., 2014	Not reported	Not reported	Not reported
Sun et al., 2015	Xi’an guanyu bio-tech company, Xi’an, China	>98	Not reported
Jin et al., 2022	Sigma-aldrich, St. Louis, MO, United States of America	Not reported	Not reported
Wang et al., 2015	Self-extracted from *Drynaria fortunei* (name reported in the original study)	Not reported	HPLC
Rodriguez et al., 2024	Millipore Sigma, St. Louis, MO, United States of America	Not reported	Not reported
Wang et al., 2022	Not reported	Not reported	Not reported

“Not reported” indicates that the information was not explicitly provided in the original article and does not indicate absence of the characteristic. Botanical origin was not inferred for commercially purchased naringin. *Drynaria fortunei* (Kunze ex Mett.) J. Sm., as reported in the original study, is currently treated by POWO, as a synonym of the accepted name *Drynaria roosii* Nakaike (Polypodiaceae).

### 3.3 Risk of bias

Risk-of-bias assessments for all 24 included experimental cohorts are shown in Figure 2. Although no domain was explicitly classified as high risk on the basis of the information reported, the predominance of unclear-risk judgements prevents this finding from being interpreted as evidence of a low overall risk of bias. All 24 studies were rated as low risk for baseline characteristics comparability, and most were rated as low risk for incomplete outcome data (*n* = 19) and other bias (*n* = 22). However, reporting was generally insufficient to establish whether adequate methods had been used for random sequence generation, allocation concealment, random housing, random outcome assessment, and blinding of caregivers, investigators, or outcome assessors. Selective outcome reporting also could not be confidently excluded because study protocols or prespecified analysis plans were generally unavailable. Thus, the absence of explicit high-risk ratings primarily reflects insufficient methodological reporting rather than confirmed methodological rigour.

**FIGURE 2:**
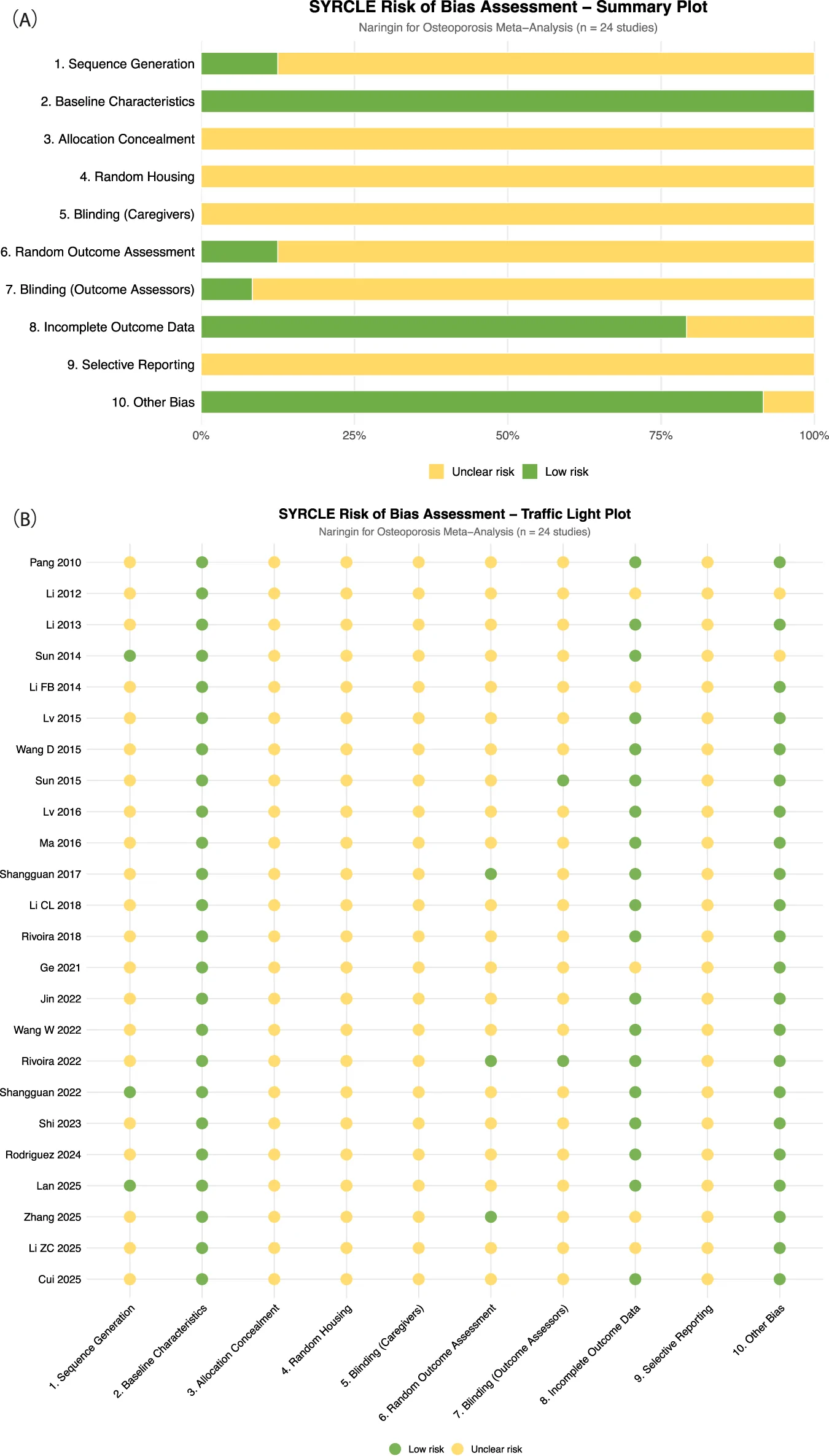
SYRCLE risk-of-bias assessment of the 24 included experimental cohorts. **(A)** Summary plot. **(B)** Traffic-light plot. Green indicates low risk and yellow indicates unclear risk.

### 3.4 Primary outcomes

#### 3.4.1 Bone mineral density (BMD)

Twenty-two experimental cohorts (*k* = 22) contributed to the BMD analysis. The pooled estimate favoured naringin for BMD compared with model control (SMD = 2.30, 95% CI 1.88 to 2.72; *I* ^2^ = 54.6%, *τ* ^2^ = 0.527; heterogeneity *p* = 0.0012; 95% PI 0.72 to 3.87; Figure 3). Substantial between-study heterogeneity was present. Positive pooled estimates were observed across the three model type categories (Figure 4A), and in both rat and mouse species (Figure 4B), with no statistically detectable between-subgroup difference in either analysis (species: χ^2^ = 1.57, df = 1, *p* = 0.210; model type: χ^2^ = 2.59, df = 2, *p* = 0.273). Pooled BMD estimates were less heterogeneous in OVX models (*I* ^2^ = 7.4%) than in the other model categories. A DL sensitivity analysis produced a materially similar pooled estimate (SMD = 2.30, 95% CI 1.88–2.72), suggesting limited sensitivity to the choice of *τ* ^2^ estimator.

**FIGURE 3:**
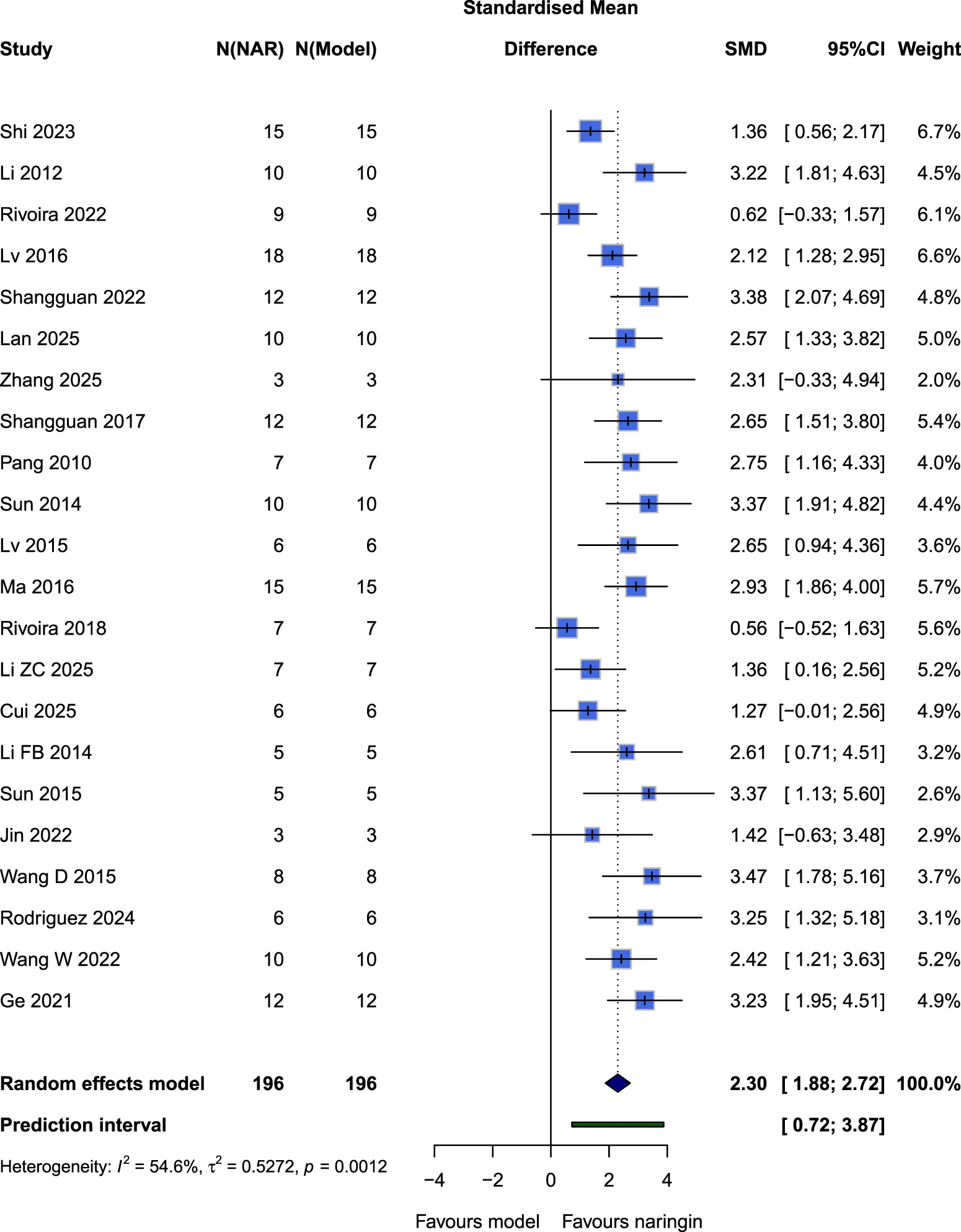
Forest plot of the effect of naringin on bone mineral density (BMD; random-effects model, REML–Knapp–Hartung). SMD >0 favours naringin. Squares and horizontal lines represent study-specific SMDs and 95% confidence intervals, with square size proportional to study weight. The diamond represents the pooled SMD. The horizontal bar below the diamond represents the 95% prediction interval. The solid vertical line indicates the null value (SMD = 0), and the dotted vertical line indicates the pooled estimate.

**FIGURE 4:**
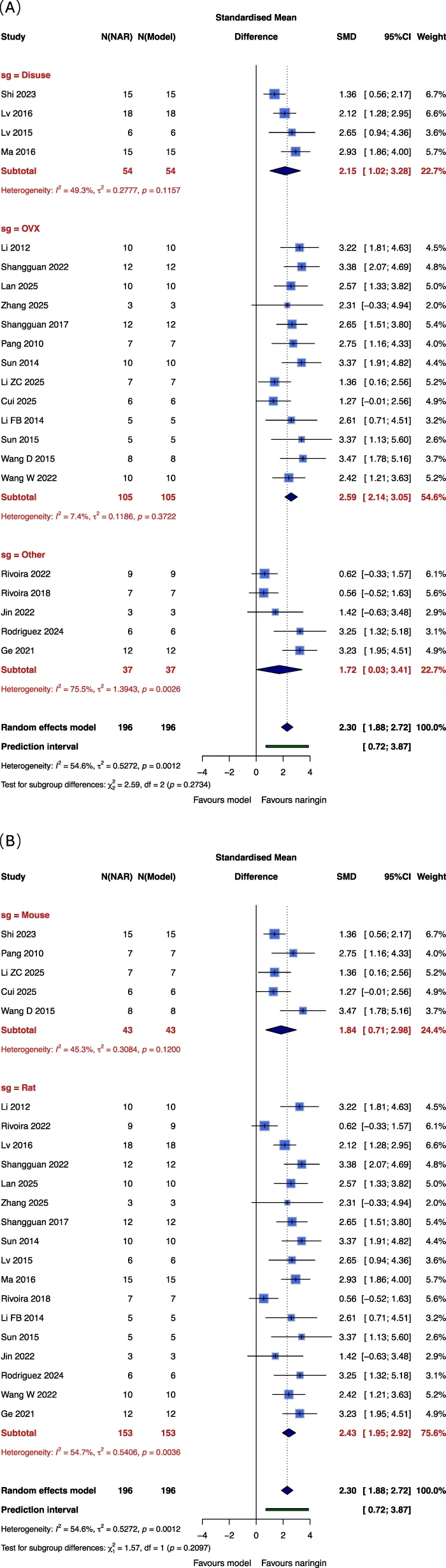
Subgroup analyses of the effect of naringin on BMD. **(A)** Exploratory subgroup analysis by osteoporosis model type (OVX, disuse, and other models). **(B)** Subgroup analysis by animal species (rat vs mouse).

#### 3.4.2 Bone volume fraction (BV/TV)

Seventeen experimental cohorts (*k* = 17) contributed to the BV/TV analysis. Naringin was associated with a significant increase in BV/TV compared with model control (SMD = 3.36, 95% CI 2.17 to 4.55, *I* ^2^ =81.6%, *τ* ^2^ =3.294; heterogeneity *p* < 0.0001; 95% PI −0.63 to 7.36; Figure 5). Considerable between-study heterogeneity was present. In the model‐type subgroup analysis (Figure 6A), a significant effect was observed in OVX models (*k* = 9, SMD = 3.56, 95% CI 2.04 to 5.07, *I* ^2^= 71.4%); in disuse models, the pooled estimate was positive but imprecise and its confidence interval included the null (*k* = 4, SMD = 4.97, 95% CI -0.94 to 10.89, *I* ^2^ = 89.3%); the other-model subgroup also showed a positive but imprecise estimate that crossed the null (*k* = 4, SMD = 1.61, 95% CI -0.86 to 4.08, *I* ^2^ = 69.4%), with no significant between-subgroup difference (χ^2^ = 5.00, df = 2, *p* = 0.082). In the species subgroup analysis ( Figure 6B), a significant effect was observed in rats (*k* = 14, SMD = 3.48, 95% CI 1.99 to 4.97, *I* ^2^ = 83.8%); in mice, the pooled estimate was positive but imprecise and did not reach statistical significance (*k* = 3, SMD = 2.95, 95% CI ‐0.33 to 6.24, *I* ^2^ = 65.4%), with no significant between-subgroup difference (χ^2^ = 0.26, df = 1, *p* = 0.608).Post-hoc sensitivity analysis excluding two studies with extreme effect sizes yielded SMD = 2.90 (95% CI 2.00 to 3.79, *I* ^2^ = 77.9%; Supplementary 3 Figure S1A). The DL sensitivity analysis produced a materially similar estimate (SMD = 3.33, 95% CI 2.39 to 4.27), supporting stability across the two tau-estimation methods.

**FIGURE 5:**
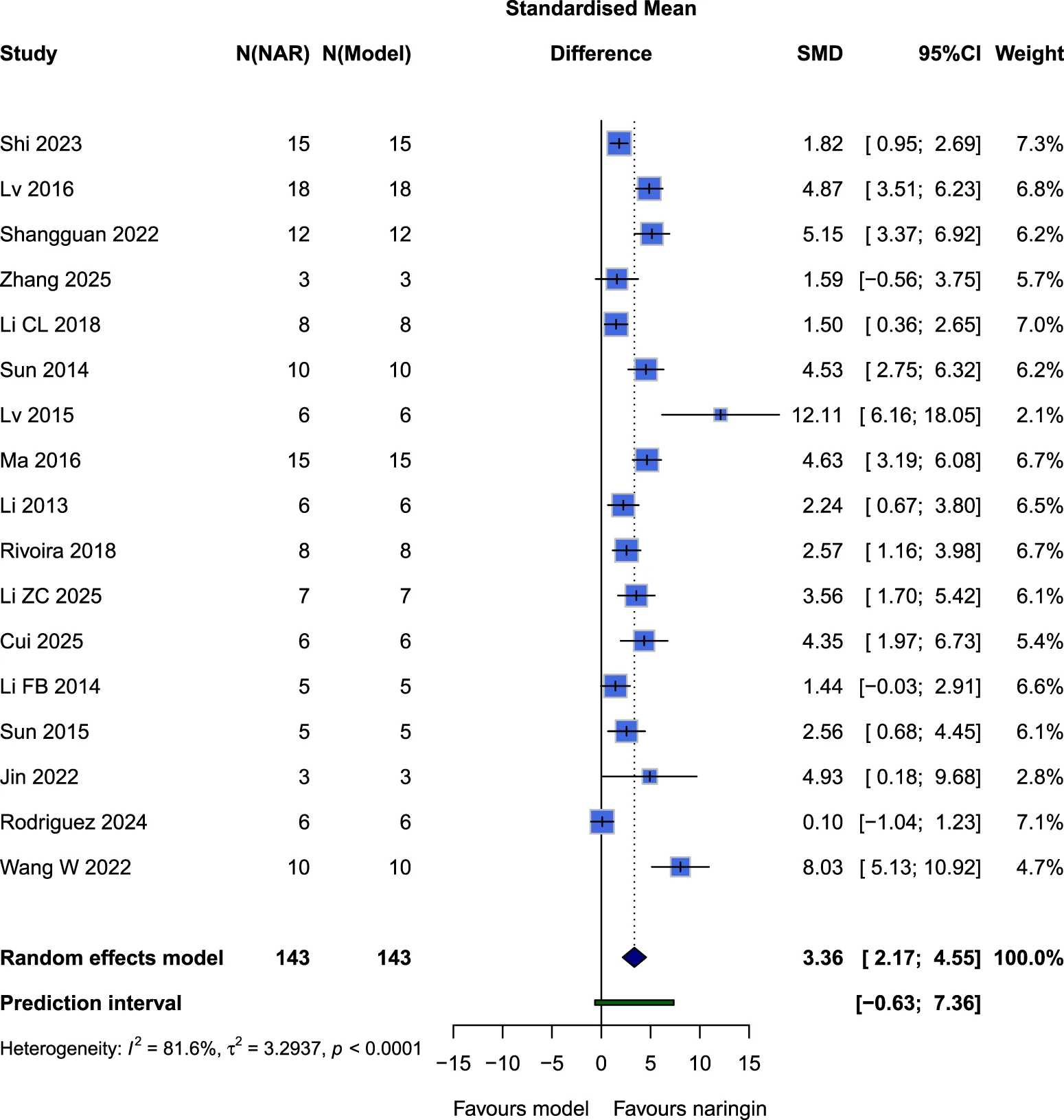
Forest plot of the effect of naringin on BV/TV. SMD >0 favours naringin.

**FIGURE 6:**
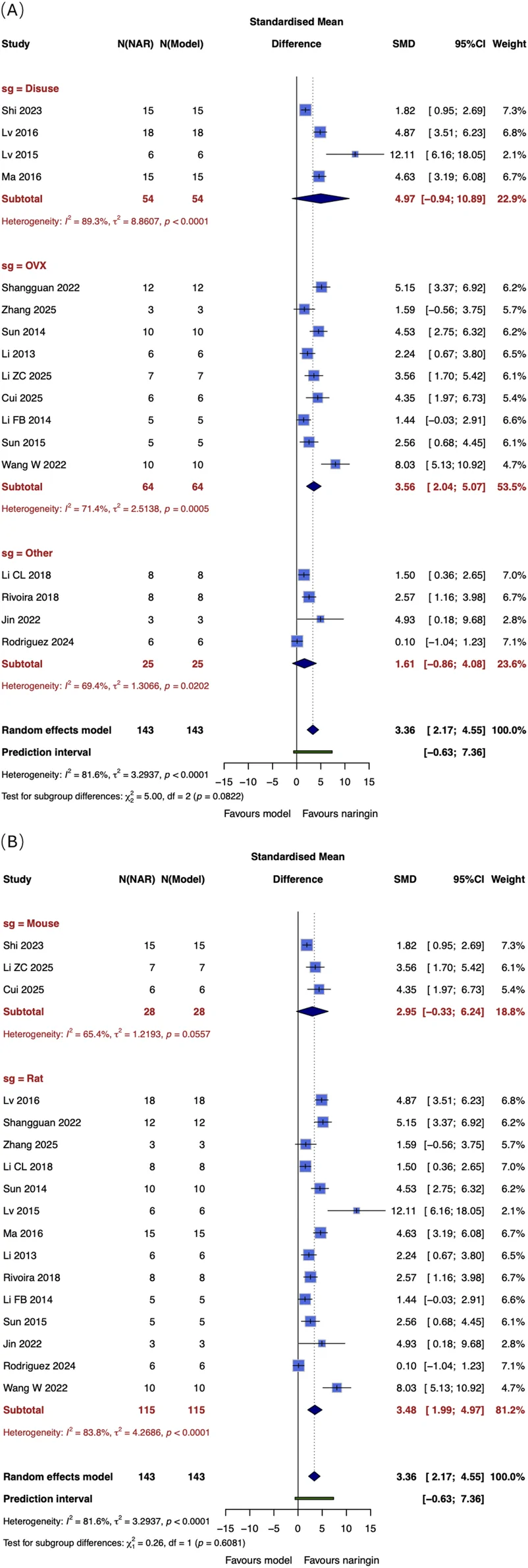
Subgroup analyses of the effect of naringin on BV/TV. **(A)** Exploratory subgroup analysis by osteoporosis model type. **(B)** Subgroup analysis by animal species (rat vs. mouse).

### 3.5 Secondary outcomes

#### 3.5.1 Trabecular microarchitecture (Tb.Th, Tb.N, and Tb.Sp)

Seventeen experimental cohorts contributed to the Tb.Th analysis. Naringin was associated with a significant increase in Tb.Th (SMD = 2.35, 95% CI 1.49 to 3.21, *I* ^2^ = 81.0%, *τ* ^2^ = 2.181; heterogeneity *p* < 0.0001; 95% PI-0.90 to 5.60; Figure 7A). An exploratory model- type subgroup analysis (Supplementary Figure 4 S2A) identified a statistically significant subgroup interaction (χ^2^ = 11.79, df = 2, *p* = 0.0027), suggesting potential model-related variation in Tb.Th responses. The pooled estimates were higher in disuse models (*k* = 4, SMD = 3.40, 95% CI 0.74-6.06) and OVX models (*k* = 9, SMD = 2.49, 95% CI 1.26-3.72), whereas the other-model subgroup, including glucocorticoid-induced, diabetic, and metabolic models (*k* = 4, SMD = 0.72, 95% CI -0.62 to 2.05), showed a smaller estimate. However, these differences should be interpreted cautiously because the non‐OVX categories were supported by relatively few studies.

**FIGURE 7:**
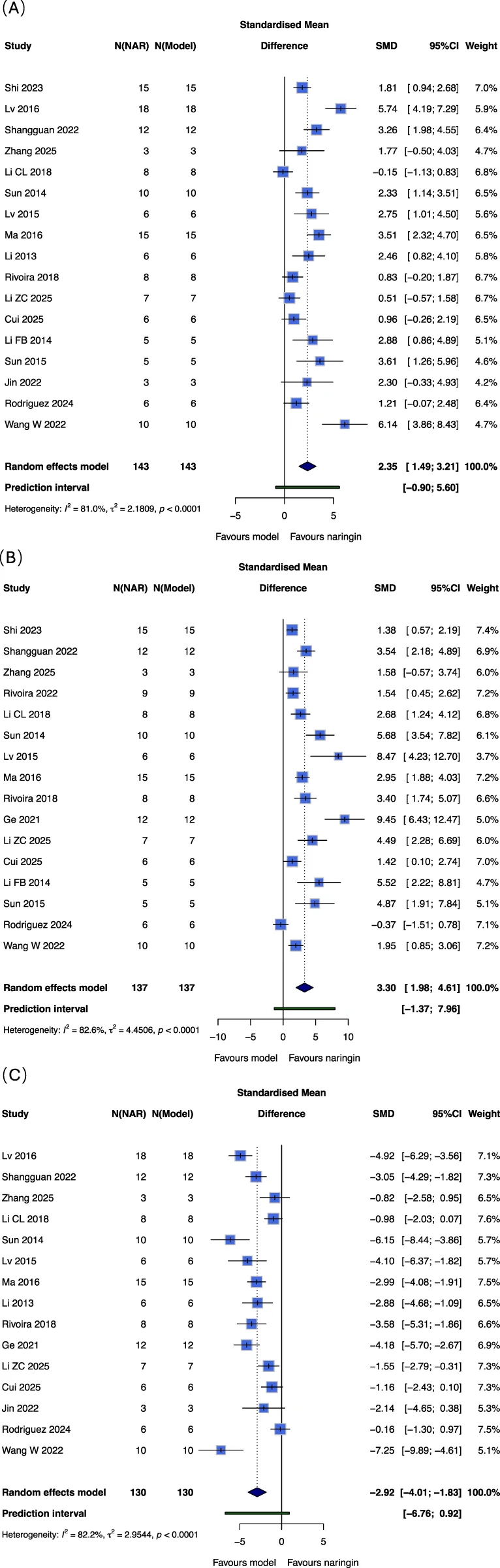
Forest plots of the effect of naringin on trabecular microarchitecture. **(A)** Trabecular thickness (Tb.Th), SMD >0 favours naringin. **(B)** Trabecular number (Tb.N), SMD >0 favours naringin. **(C)** Trabecular separation (Tb.Sp), SMD <0 favours naringin.

Sixteen experimental cohorts contributed to the Tb.N analysis. Naringin was associated with a significant increase in Tb.N (SMD = 3.30, 95% CI 1.98 to 4.61, *p* < 0.0001; *I* ^2^ = 82.6%, *τ* ^2^ = 4.451; heterogeneity *p* < 0.0001; 95% PI -1.37 to 7.96; Figure 7B). An exploratory model-type subgroup analysis analysis (Supplementary Figure 4 S2B) showed no significant between-subgroup difference (χ^2^ = 0.06, df = 2, *p* = 0.971), and the available data did not provide evidence that model type explained the observed heterogeneity. Post-hoc sensitivity analysis excluding two studies with extreme effect sizes yielded SMD = 2.63 (95% CI 1.65 to 3.61, *I* ^2^ = 76.7%; Supplementary 3, Figure S1B).

Fifteen experimental cohorts contributed to the Tb. Sp analysis. Naringin was associated with a significant reduction in Tb. Sp (SMD = −2.92, 95% CI −4.01 to −1.83; *I* ^2^ = 82.2%, *τ* ^2^ = 2.954; heterogeneity *p* < 0.0001; 95% PI ‐6.76 to 0.92; Figure 7C). A negative SMD indicates a favourable reduction in inter-trabecular spacing. An exploratory model-type subgroup analysis (Supplementary 4, Figure S2C) showed no significant between-subgroup difference (χ^2^ = 3.33, df = 2, *p* = 0.189), and the available data did not provide evidence that model type explained the observed heterogeneity.

#### 3.5.2 Bone formation-related markers (OCN and P1NP)

Twelve experimental cohorts contributed to the OCN analysis. Naringin was associated with a significant increase in serum OCN (SMD = 2.11, 95% CI 1.21 to 3.01; *I* ^2^ = 80.5%, *τ* ^2^ = 1.538, heterogeneity *p* < 0.0001; 95% PI ‐0.77 to 4.99; Figure 8A). One study (Shi et al., 2023) used a disuse model but was the sole contributor to that subgroup category and was therefore reported narratively (SMD = 1.87). The remaining 11 cohorts were pooled across OVX (*k* = 8, SMD = 1.90, 95% CI 0.61 to 3.19, I2 = 85.7%) and other models (*k* = 3, SMD = 2.62, 95% CI 0.22 to 5.03, I2 = 33.9%), with no significant between-subgroup difference (χ^2^ = 0.85, df = 1, *p* = 0.356; Supplementary 4, Figure S2D).

Seven experimental cohorts contributed to the P1NP analysis. Naringin was associated with a significant increase in serum P1NP (SMD = 2.32, 95% CI 0.82 to 3.83, *I* ^2^ = 78.5%, *τ* ^2^ = 1.988; heterogeneity *p* < 0.0001; 95% PI −1.42 to 6.07; Figure 8B). Considerable heterogeneity was observed, and the wide prediction interval crossing zero indicates uncertainty about the magnitude of effect across settings.

**FIGURE 8:**
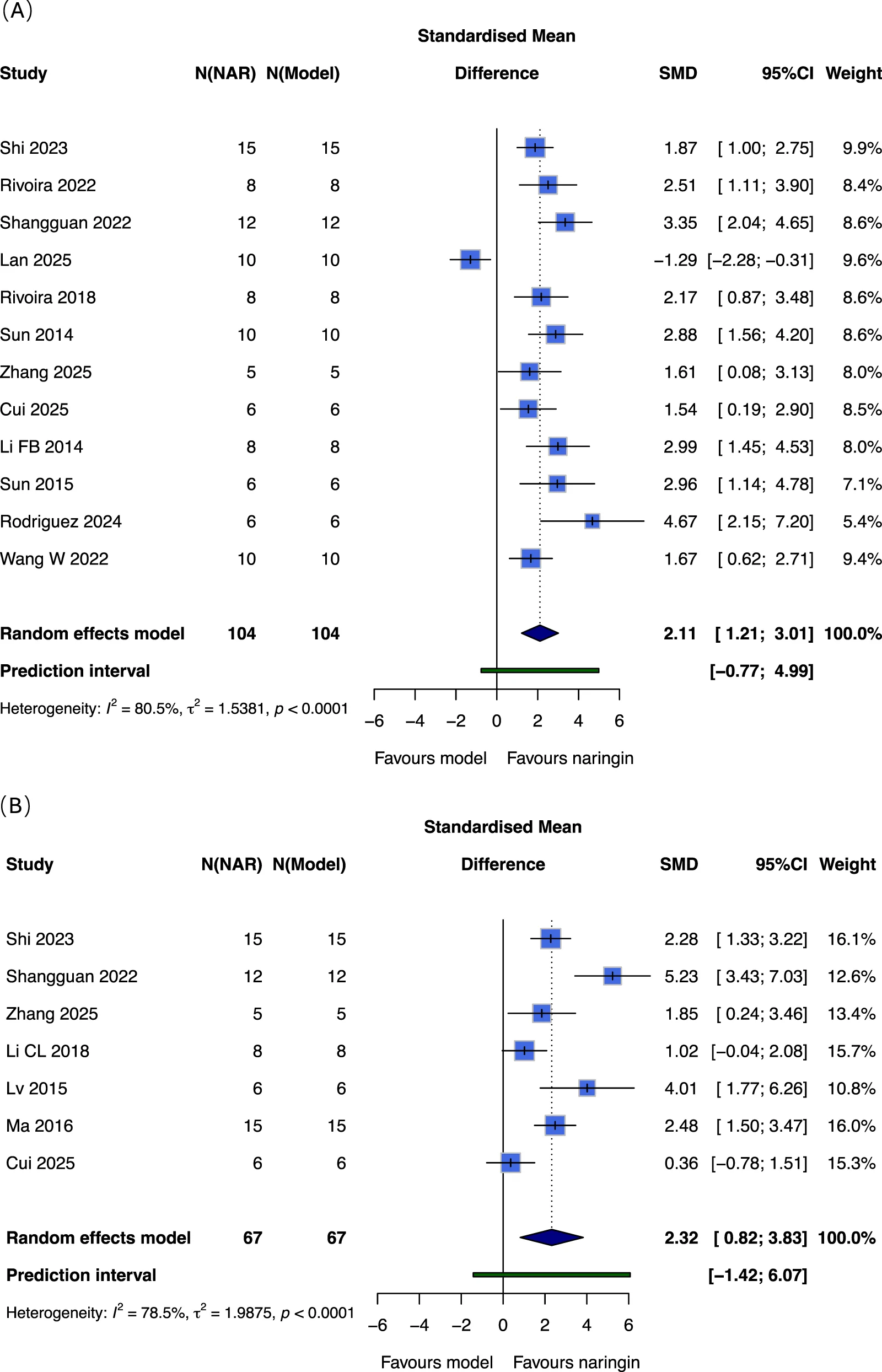
Forest plots of the effect of naringin on bone formation-related markers. **(A)** Osteocalcin (OCN), SMD >0 favours naringin. **(B)** P1NP, SMD >0 favours naringin.

#### 3.5.3 CTX-1, TRAP, and ALP

Nine experimental cohorts contributed to the CTX-1 analysis. Naringin was associated with a significant reduction in serum CTX-1 (SMD = −2.38, 95% CI −2.97 to −1.79; *I* ^2^ = 28.8%, *τ* ^2^ = 0.025; heterogeneity *p* = 0.188; 95% PI −3.00 to −1.76; Figure 9A). This was the only secondary outcome with moderate heterogeneity; the prediction interval entirely excluded zero, indicating a relatively consistent pattern of reduced bone resorption marker levels across study settings.

**FIGURE 9:**
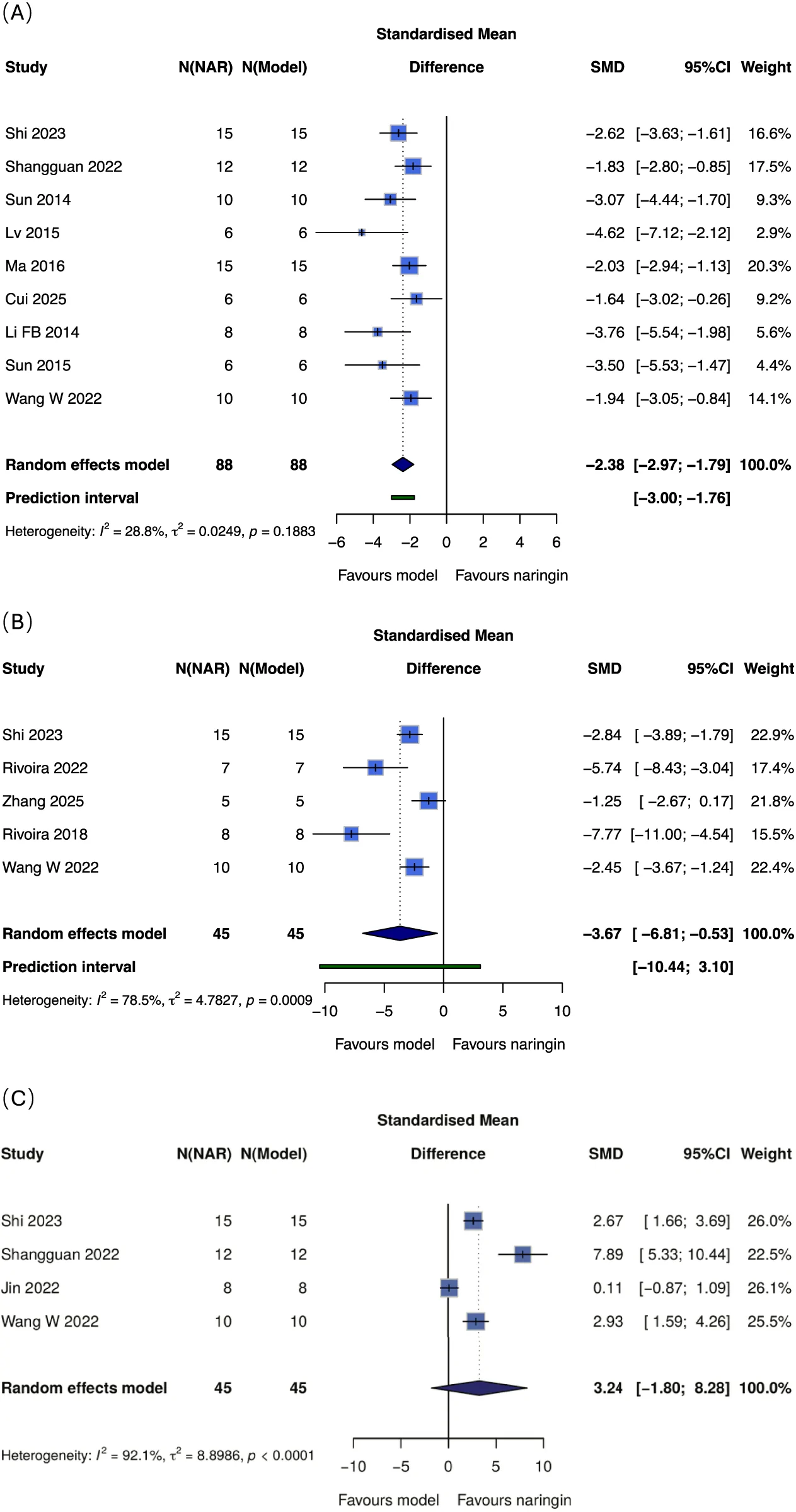
Forest plots of the effects of naringin on CTX-1, TRAP, and ALP. **(A)** CTX-1, SMD <0 favours naringin. **(B)** TRAP, SMD <0 favours naringin. **(C)** ALP.

Five experimental cohorts contributed to the TRAP analysis. The pooled mean estimate favoured a reduction in serum TRAP (SMD = −3.67, 95% CI −6.81 to *I* ^2^ = 78.5%, *τ* ^2^ = 4.783; heterogeneity *p* = 0.0009; Figure 9B). However, the evidence base was small, heterogeneity was considerable, and the prediction interval was wide and crossed the null. In leave-one-out sensitivity analyses, the direction of the pooled estimate remained negative, but statistical significance was not retained in three of five iterations. The TRAP result should therefore be interpreted as suggestive and highly uncertain rather than as robust evidence of reduced bone resorption.

Four experimental cohorts contributed to the ALP analysis. The pooled estimate did not provide reliable evidence of an effect on serum ALP (SMD = 3.24, 95% CI −1.80 to 8.28; *I* ^2^ = 92.1%, *τ* ^2^ = 8.899; heterogeneity *p* < 0.0001; Figure 9C). A prediction interval was not estimated because fewer than five studies contributed.

### 3.6 Dose–response meta-regression

Exploratory multilevel dose–response meta-regression was performed using all eligible dose-arm comparisons while accounting for dependence arising from shared controls. Seven outcomes had at least 10 eligible comparisons (Table 5). On the raw-dose scale, dose was positively associated with BMD (slope = 0.0068 per mg/kg/day, 95% CI 0.0048–0.0089) and Tb.N (slope = 0.0152, 95% CI 0.0072–0.0232), and inversely associated with CTX-1 (slope = −0.0088, 95% CI −0.0162 to −0.0013). No significant associations were observed for BV/TV, Tb.Th, Tb. Sp, or OCN.

On the log10-dose scale, positive associations were observed for BMD (slope = 1.38 per ten-fold dose increase, 95% CI 0.31–2.46), BV/TV (slope = 3.59, 95% CI 1.34–5.84), Tb.Th (slope = 2.52, 95% CI 0.70–4.35), and Tb.N (slope = 5.40, 95% CI 3.58–7.22), whereas CTX-1 showed an inverse association (slope = −2.64, 95% CI −4.61 to −0.67). Associations for Tb. Sp and OCN were non-significant. Complete model estimates are presented in Table 5, and the log10-dose relationships are shown in Figure 10. Because the raw- and log10-dose models produced different significance patterns and may be confounded by species, osteoporosis model, administration route, and treatment duration, these ecological analyses neither establish a causal dose‐response relationship nor identify an optimal dose.

**FIGURE 10:**
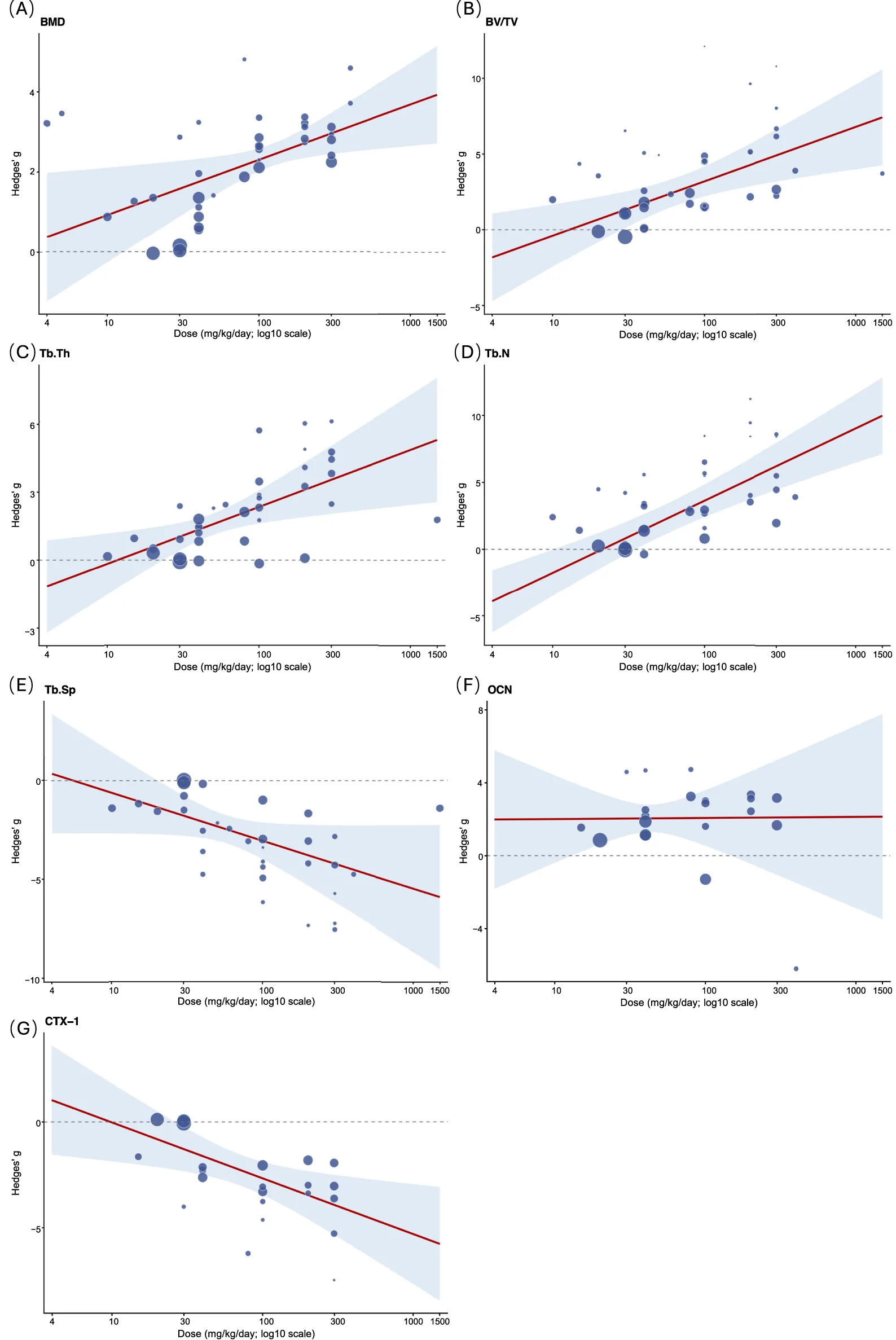
Exploratory multilevel meta-regression of log10-transformed naringin dose and SMD using all eligible dose-arm comparisons. Panels show **(A)** BMD, **(B)** BV/TV, **(C)** Tb.Th, **(D)** Tb.N, **(E)** Tb. Sp, **(F)** OCN, and **(G)** CTX-1. The regression slope represents the estimated change in SMD associated with a ten-fold increase in naringin dose. Bubble size is proportional to the inverse sampling variance; solid red lines indicate fitted meta-regression relationships, and shaded areas indicate 95% confidence bands.

Influence diagnostics identified several potentially influential dose arms or study clusters, but none was consistently influential across outcomes and model specifications (Tables 6, 7). Excluding doses ≥300 mg/kg/day or the outcome-specific maximum dose changed some coefficients and significance classifications, indicating sensitivity to sparse observations at the upper end of the dose range. For CTX-1, both exclusions removed the 300 mg/kg/day comparisons and reduced the analysis to eight independent studies. Overall, the sensitivity analyses did not support a stable monotonic dose–response relationship.

Only BMD met the feasibility criterion for multivariable analysis, comprising 22 study clusters and 41 dose-arm effects. After adjustment for species, its association with dose remained significant on both the raw scale (slope = 0.0069, 95% CI 0.0049–0.0089) and log10 scale (slope = 1.51, 95% CI 0.27–2.76). Species was not independently associated with BMD in either model, and significant residual heterogeneity remained (both *p* < 0.001). No other outcome met the requirements for dose-plus-species modelling, and none supported dose-plus-model-type analysis without a substantial risk of overfitting.

### 3.7 Three-dimensional dose–duration visualization

Three-dimensional plots descriptively displayed dose, treatment duration, and study identity for the five structural outcomes with at least 10 studies (Figure 11). Because multiple arms from the same study were non-independent, their colour-coded nominal p-value categories were not interpreted inferentially; dose-response inference relied on the multilevel models in Table 5 and Figure 10.

**FIGURE 11:**
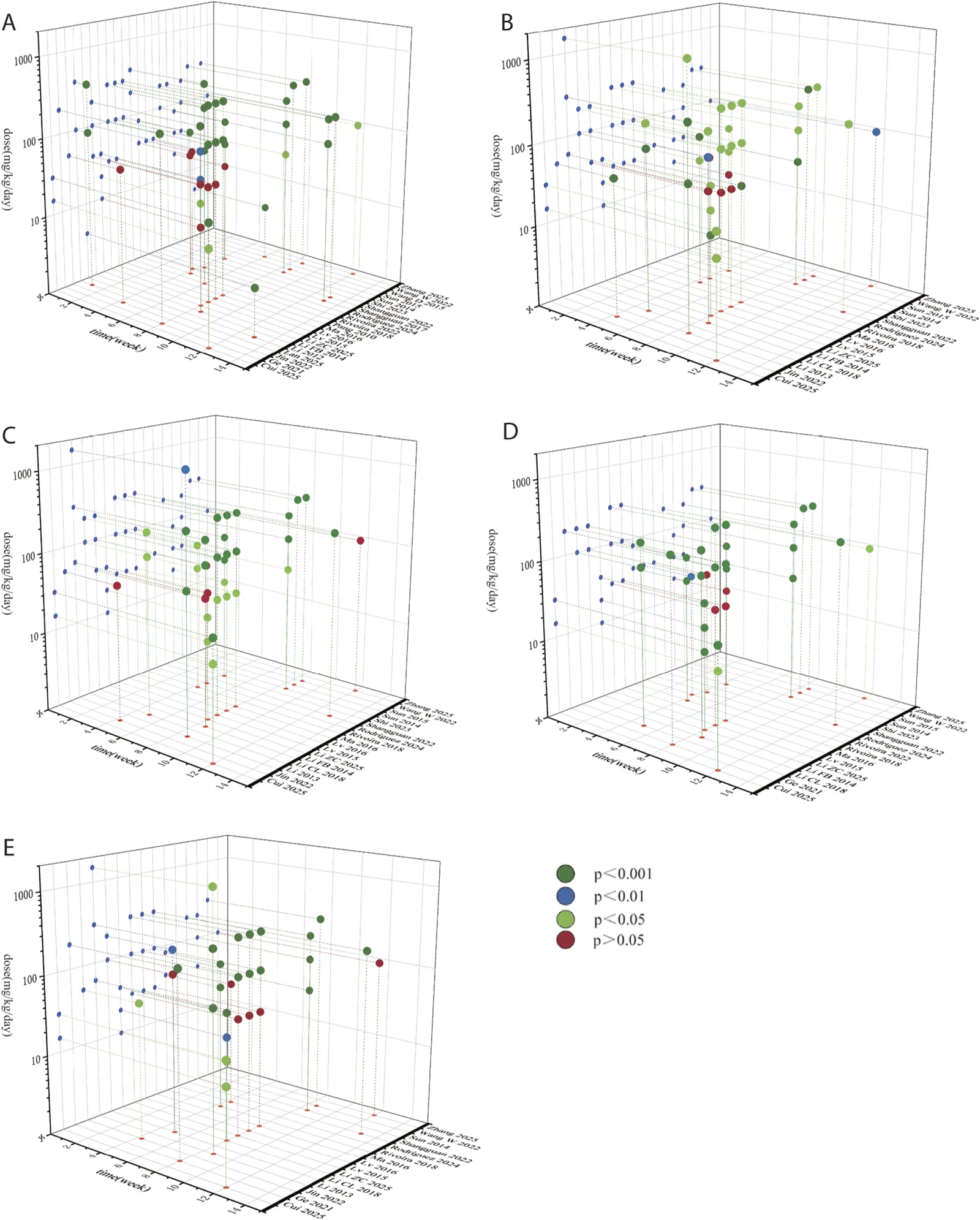
Descriptive three-dimensional plots of naringin dose, treatment duration, and study identity across all reported dose arms. **(A)** BMD; **(B)** BV/TV; **(C)** Tb.Th; **(D)** Tb.N; and **(E)** Tb. Sp. Colours denote nominal *p*-value categories only; dose arms from the same study are non-independent and were not interpreted inferentially for dose–response trend.

### 3.8 Sensitivity analyses and assessment of small-study effects

Leave-one-out analyses showed that pooled-effect directions remained stable for all outcomes with *k* ≥ 5, with statistical significance retained in all iterations except for TRAP, as described in Section 3.5.3.

The primary, highest-dose-arm, and all-dose-arm multilevel analyses were compared (Table 4). Replacing the prespecified representative dose with the highest available dose did not change the direction or significance of any pooled outcome (Table 8; Figure 12). However, study-level dose meta-regression was sensitive to dose-arm selection: the dose associations for BMD, Tb.Th, and Tb. Sp became non-significant when the highest-dose arm was used, whereas OCN showed an inverse association (representative-dose baseline results in Supplementary 2, Table S4). These dose–response findings should therefore be considered exploratory.

**TABLE 4:** Comparison of the primary, highest-dose-arm, and all-dose-arm multilevel sensitivity analyses.

Outcome	Independent studies	Primary analysis SMD (95% CI)	Highest-dose analysis SMD (95% CI)	All-dose-arm comparisons	All-dose-arm multilevel analysis SMD (95% CI)	All-dose-arm prediction interval	Pooled interpretation changed
BMD	22	2.30 (1.88–2.72)	2.74 (2.33–3.15)	41	2.12 (1.74–2.51)	−0.07 to 4.32	No
BV/TV	18	3.36 (2.17–4.55)	4.40 (3.05–5.74)	38	3.06 (2.36–3.76)	−1.21 to 7.33	No
Tb.Th	18	2.35 (1.49–3.21)	2.98 (2.08–3.88)	37	2.13 (1.51–2.75)	−1.12 to 5.38	No
Tb.N	18	3.30 (1.98–4.61)	4.52 (3.01–6.02)	36	3.40 (2.53–4.27)	−1.38 to 8.17	No
Tb.Sp	16	−2.92 (−4.01 to −1.83)	−3.58 (−4.78 to −2.38)	32	−2.92 (−3.76 to −2.11)	−6.58 to 0.72	No
OCN	12	2.11 (1.21–3.01)	2.42 (0.65–4.20)	21	2.06 (0.86–3.26)	−1.89 to 6.01	No
ALP	4	3.24 (−1.80–8.28)	3.53 (−1.49–8.56)	6	3.01 (−1.52–7.55)	−3.35 to 9.37	No
P1NP	7	2.32 (0.82–3.83)	3.46 (1.93–4.99)	18	2.32 (1.43–3.21)	−1.33 to 5.97	No
CTX-1	10	−2.38 (−2.97 to −1.79)	−3.66 (−4.83 to −2.49)	23	−2.55 (−2.96 to −2.14)	−5.98 to 0.89	No
TRAP	5	−3.67 (−6.81 to −0.53)	−3.61 (−6.09 to −1.13)	8	−3.47 (−6.32 to −0.63)	−8.39 to 1.44	No

**FIGURE 12:**
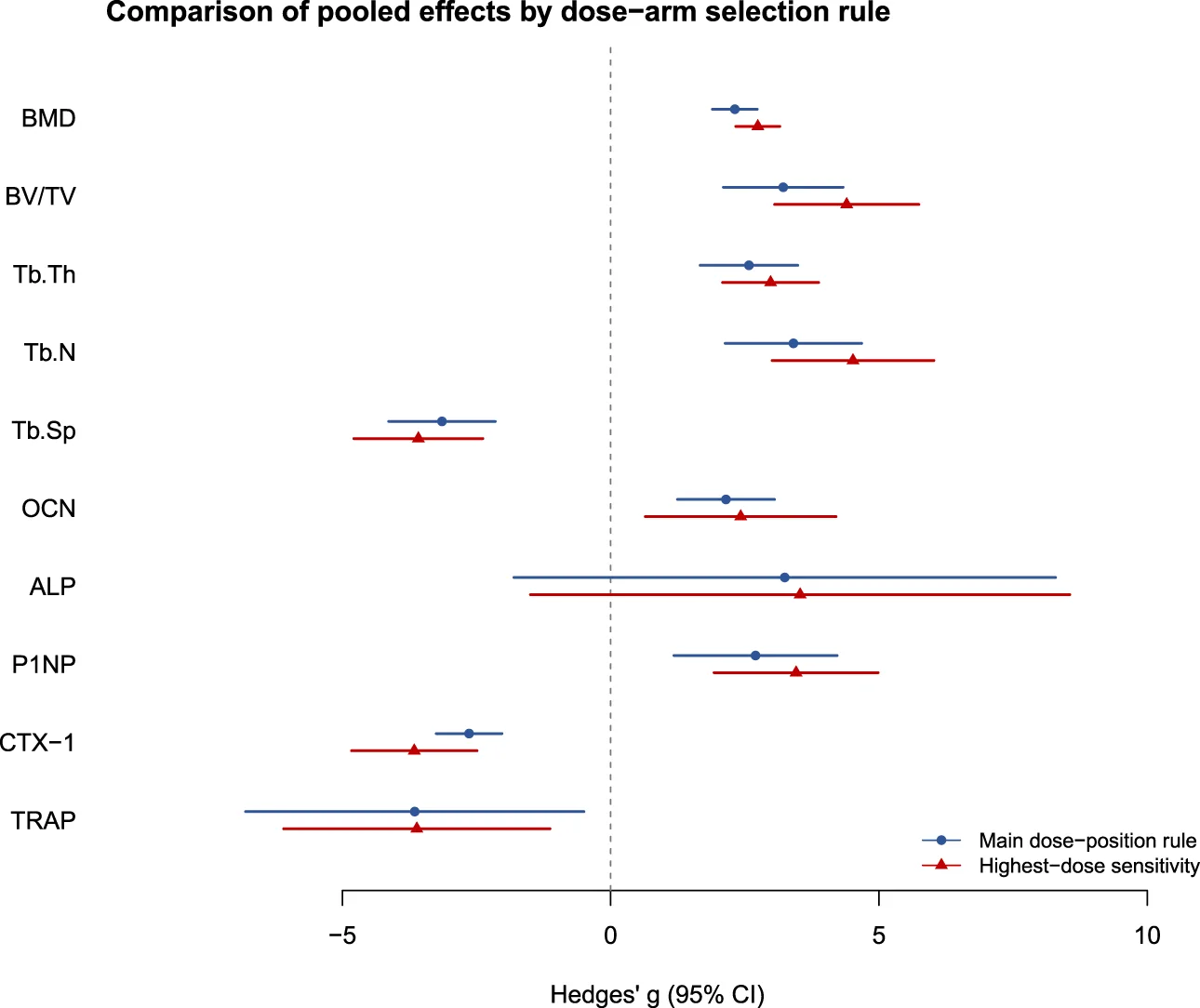
Comparison of pooled effects under the prespecified representative-dose-position rule and the highest-dose-arm sensitivity analysis. Points represent SMD, and horizontal lines represent 95% confidence intervals.

Similarly, multilevel models retaining all eligible dose arms produced pooled estimates with unchanged directions and significance classifications (Table 8). However, prediction intervals for BMD and CTX-1, which included zero in the primary analyses, included zero in the all-dose-arm models, indicating greater uncertainty about effects in future settings. Together with the variation in dose associations across model specifications (Section 3.6; Table 5), these wider prediction intervals limit conclusions regarding reproducibility despite the stability of the pooled mean effects.

**TABLE 5:** Exploratory multilevel meta-regression of raw and log10-transformed naringin dose using all eligible dose-arm comparisons.

Outcome	Studies/effects	Dose range (mg/kg/day)	Raw-dose slope (95% CI)	p value	Log10-dose slope (95% CI)	p value
BMD	22/41	4–400	0.0068 (0.0048–0.0089)	<0.001	1.38 (0.31–2.46)	0.014
BV/TV	18/38	10–1,500	0.0027 (−0.0016–0.0071)	0.197	3.59 (1.34–5.84)	0.004
Tb.Th	18/37	10–1,500	0.0017 (−0.0023–0.0058)	0.369	2.52 (0.70–4.35)	0.010
Tb.N	18/36	10–400	0.0152 (0.0072–0.0232)	0.001	5.40 (3.58–7.22)	<0.001
Tb.Sp	16/32	10–1,500	−0.0010 (−0.0051 to 0.0032)	0.626	−2.43 (−4.96 to 0.10)	0.059
OCN	12/21	15–400	−0.0053 (−0.0205 to 0.0100)	0.460	0.06 (−3.55–3.67)	0.971
CTX-1	10/23	15–300	−0.0088 (−0.0162 to −0.0013)	0.026	−2.64 (−4.61 to −0.67)	0.015

Values are meta-regression slopes with 95% CIs. Raw-dose and log10-dose slopes represent SMD, changes per 1 mg/kg/day and ten-fold dose increase, respectively. “Studies/effects” indicates independent studies and dose-arm effect estimates. Multilevel random-effects models accounted for shared-control dependence.

**TABLE 6:** Influence diagnostics and high-dose exclusion sensitivity analyses for multilevel dose meta-regression models. Panel a. Influence diagnostics.

Outcome	Dose scale	Top cook study	Cook’s distance	Highest-leverage effect	Dose	Hat value	Largest-residual effect	Dose	Studentized residual
BMD	Raw	Lv 2016	0.242	Lan 2025_2	400	0.143	Rivoira 2018_2	80	2.124
BMD	Log10	Li 2012	2.520	Li 2012_1	4	0.173	Li 2012_1	4	3.279
BV/TV	Raw	Li/Xu 2013	50.831	Li/Xu 2013_3	1,500	0.819	Li/Xu 2013_3	1,500	−3.766
BV/TV	Log10	Li/Xu 2013	6.190	Li/Xu 2013_3	1,500	0.222	Lv 2015_2	100	2.532
Tb.Th	Raw	Li/Xu 2013	111.655	Li/Xu 2013_3	1,500	0.855	Li/Xu 2013_3	1,500	−5.603
Tb.Th	Log10	Li/Xu 2013	6.065	Li/Xu 2013_3	1,500	0.239	Li/Xu 2013_3	1,500	−3.346
Tb.N	Raw	Lan 2025	0.778	Lan 2025_2	400	0.244	Song 2014_3	200	2.542
Tb.N	Log10	Lv 2016	1.420	Ma 2016_3	300	0.142	Wang 2022_1	300	−2.130
Tb.Sp	Raw	Li/Xu 2013	91.763	Li/Xu 2013_3	1,500	0.857	Li/Xu 2013_3	1,500	5.052
Tb.Sp	Log10	Li/Xu 2013	5.977	Li/Xu 2013_3	1,500	0.270	Li/Xu 2013_3	1,500	3.700
OCN	Raw	Lan 2025	4.844	Lan 2025_2	400	0.353	Lan 2025_2	400	−5.631
OCN	Log10	Lan 2025	1.730	Cui 2025_1	15	0.199	Lan 2025_2	400	−6.552
CTX-1	Raw	Wang 2022	0.474	Ma 2016_3	300	0.181	Shi 2023_3	80	−2.840
CTX-1	Log10	Cui 2025	0.705	Cui 2025_1	15	0.165	Shi 2023_3	80	−2.848

Cook’s distance was calculated at the study-cluster level, whereas studentized residuals and hat values were assessed at the dose-arm level. High-dose exclusions were exploratory sensitivity analyses. CI, confidence interval.

**TABLE 7:** Panel b. High-dose exclusion sensitivity analyses.

Outcome	Dose scale	Sensitivity specification	Studies/effects	Remaining dose range (mg/kg/day)	Slope (95% CI)	p value	Significance changed vs. baseline
BMD	Raw	Exclude ≥300 mg/kg/day	20/34	4–200	0.0119 (0.0069–0.0170)	<0.001	No
BMD	Raw	Exclude maximum dose	22/39	4–300	0.0067 (0.0042–0.0092)	<0.001	No
BMD	Log10	Exclude ≥300 mg/kg/day	20/34	4–200	1.28 (−0.18–2.74)	0.083	Yes
BMD	Log10	Exclude maximum dose	22/39	4–300	1.25 (0.16–2.35)	0.027	No
BV/TV	Raw	Exclude ≥300 mg/kg/day	16/30	10–200	0.0271 (0.0095–0.0447)	0.005	Yes
BV/TV	Raw	Exclude maximum dose	18/37	10–400	0.0122 (0.0043–0.0201)	0.005	Yes
BV/TV	Log10	Exclude ≥300 mg/kg/day	16/30	10–200	5.10 (2.51–7.69)	<0.001	No
BV/TV	Log10	Exclude maximum dose	18/37	10–400	4.41 (2.46–6.35)	<0.001	No
Tb.Th	Raw	Exclude ≥300 mg/kg/day	16/30	10–200	0.0189 (0.0058–0.0319)	0.008	Yes
Tb.Th	Raw	Exclude maximum dose	18/36	10–300	0.0126 (0.0082–0.0170)	<0.001	Yes
Tb.Th	Log10	Exclude ≥300 mg/kg/day	16/30	10–200	3.72 (1.77–5.67)	0.001	No
Tb.Th	Log10	Exclude maximum dose	18/36	10–300	3.43 (2.29–4.56)	<0.001	No
Tb.N	Raw	Exclude ≥300 mg/kg/day	16/30	10–200	0.0331 (0.0163–0.0499)	<0.001	No
Tb.N	Raw	Exclude maximum dose	18/35	10–300	0.0178 (0.0090–0.0267)	<0.001	No
Tb.N	Log10	Exclude ≥300 mg/kg/day	16/30	10–200	5.94 (3.39–8.49)	<0.001	No
Tb.N	Log10	Exclude maximum dose	18/35	10–300	5.46 (3.48–7.43)	<0.001	No
Tb.Sp	Raw	Exclude ≥300 mg/kg/day	15/25	10–200	−0.0173 (−0.0296 to −0.0050)	0.009	Yes
Tb.Sp	Raw	Exclude maximum dose	16/31	10–400	−0.0120 (−0.0185 to −0.0054)	0.002	Yes
Tb.Sp	Log10	Exclude ≥300 mg/kg/day	15/25	10–200	−3.48 (−5.58 to −1.37)	0.003	Yes
Tb.Sp	Log10	Exclude maximum dose	16/31	10–400	−3.60 (−5.37 to −1.83)	<0.001	Yes
OCN	Raw	Exclude ≥300 mg/kg/day	10/18	15–200	0.0056 (−0.0014–0.0126)	0.103	No
OCN	Raw	Exclude maximum dose	12/20	15–300	0.0029 (−0.0025–0.0083)	0.258	No
OCN	Log10	Exclude ≥300 mg/kg/day	10/18	15–200	3.03 (1.99–4.08)	<0.001	Yes
OCN	Log10	Exclude maximum dose	12/20	15–300	2.73 (1.80–3.66)	<0.001	Yes
CTX-1	Raw	Exclude ≥300 mg/kg/day	8/18	15–200	−0.0115 (−0.0255 to 0.0026)	0.093	Yes
CTX-1	Raw	Exclude maximum dose	8/18	15–200	−0.0115 (−0.0255 to 0.0026)	0.093	Yes
CTX-1	Log10	Exclude ≥300 mg/kg/day	8/18	15–200	−2.85 (−5.61 to −0.09)	0.045	No
CTX-1	Log10	Exclude maximum dose	8/18	15–200	−2.85 (−5.61 to −0.09)	0.045	No

**TABLE 8:** Sensitivity analyses assessing the robustness of pooled effects of naringin in animal models of osteoporosis.

Outcome	Sensitivity analysis	Purpose	Key finding	Interpretation
BMD	Highest-dose arm sensitivity analysis	Evaluate influence of representative dose selection	Direction and statistical significance remained unchanged	Primary conclusion was robust
BMD	Alternative tau estimator sensitivity analysis	Evaluate influence of variance estimation approach	Pooled estimate remained materially similar	Results were stable across modelling approaches
BMD	All-dose-arm multilevel model accounting for shared controls	Assess dependence from multiple dose arms	Mean effect remained favourable; prediction interval included the null	Effect signal remained, but reproducibility was uncertain
BMD	Dose meta-regression under alternative dose selection	Assess robustness of dose association	Association varied according to dose modelling strategy	Dose–response findings remain exploratory
BV/TV	Extreme-effect-size exclusion sensitivity analysis	Assess influence of potential outliers	Favourable effect remained after exclusion	Main conclusion was unchanged
BV/TV	Highest-dose arm sensitivity analysis	Evaluate influence of dose-arm selection	Direction and significance remained unchanged	Pooled estimate was robust
Tb.Th/Tb.N	Extreme-effect-size exclusion sensitivity analysis	Assess influence of influential estimates	Favourable direction was maintained	Findings remained consistent
Tb.Sp	Highest-dose arm sensitivity analysis	Evaluate influence of dose-arm selection	Direction and significance remained unchanged	Pooled effect was robust
OCN	Alternative dose modelling and dose-arm sensitivity analyses	Assess stability of dose-related findings	Dose associations differed across analytical specifications	Dose interpretation requires caution
CTX-1	All-dose-arm multilevel model	Assess shared-control dependence	Direction and statistical significance maintained	Evidence was relatively consistent
TRAP	Leave-one-out sensitivity analysis	Assess influence of individual studies	Direction remained favourable, but significance was lost in several iterations	Evidence remained uncertain
Structural outcomes	Egger tests and funnel plot assessment	Assess potential small-study effects	Small-study effects were suggested for several outcomes	Potential inflation of pooled estimates cannot be excluded
Multiple outcomes	Trim-and-fill sensitivity analysis	Explore influence of funnel asymmetry	Effect direction remained after imputation	Interpretation depends on model assumptions

Funnel plots and Egger’s tests indicated potential asymmetry for BMD (*p* = 0.0277), BV/TV (*p* = 0.0077), Tb.Th (*p* = 0.0282), Tb.N (*p* = 0.0010), and Tb. Sp (*p* = 0.0342), but not OCN (p = 0.0512;Figure 13). CTX-1 was not assessed because fewer than 10 studies contributed to the primary analysis. Given the mathematical association between SMD and its standard error, the observed asymmetry was interpreted as potential small-study effects rather than definitive publication bias.

**FIGURE 13:**
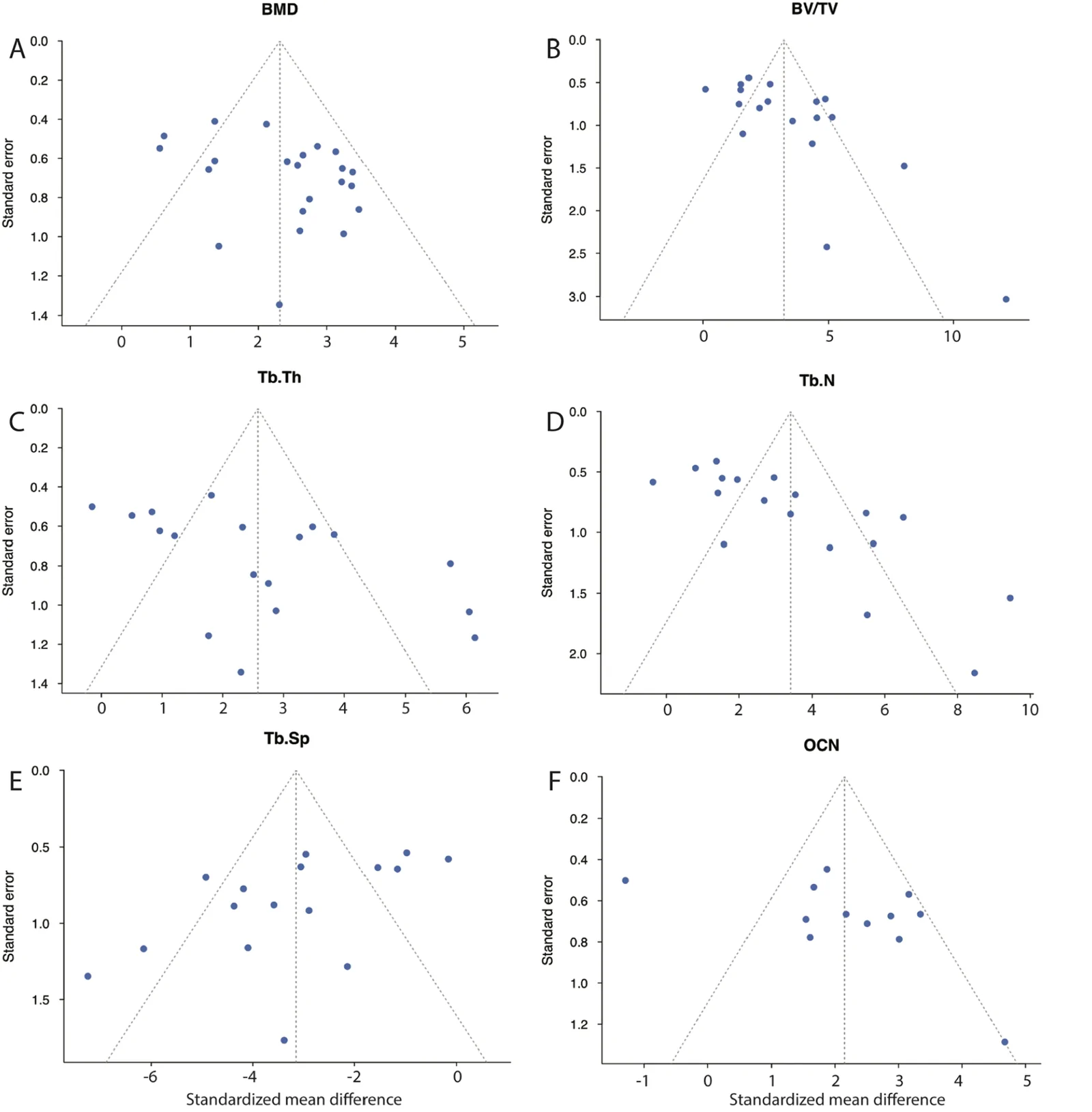
Funnel plots for outcomes with at least 10 contributing studies. **(A)** BMD (Egger p = 0.0277); **(B)** BV/TV (p = 0.0077); **(C)** Tb.Th (p = 0.0282); **(D)** Tb.N (p = 0.0010); **(E)** Tb. Sp (p = 0.0342); and **(F)** OCN (p = 0.0512).

Trim-and-fill analyses imputed four to seven hypothetical studies and reduced the absolute pooled estimates by 8.7% for BMD, 30.4% for BV/TV, 42.1% for Tb.Th, 41.2% for Tb.N, 32.1% for Tb. Sp, and 37.0% for OCN (Table 9). All adjusted estimates retained their original direction and 95% CIs excluding zero. Nevertheless, the reduced magnitudes suggest possible overestimation. Because trim-and-fill results depend on assumptions about funnel-plot asymmetry and substantial heterogeneity remained, they should be treated as sensitivity estimates rather than corrected true effects or evidence of reproducibility.

**TABLE 9:** Trim-and-fill sensitivity analyses for potential small-study effects.

Outcome	k	Egger p value	Primary SMD (95% CI)	Model-imputed studies (direction)	Trim-and-fill SMD (95% CI)	Change in absolute magnitude (%)	Significance changed
BMD	22	0.0277	2.30 (1.88–2.72)	4 (left)	2.10 (1.68–2.52)	8.7	No
BV/TV	17	0.0077	3.36 (2.17–4.55)	5 (left)	2.34 (0.91–3.77)	30.4	No
Tb.Th	17	0.0282	2.35 (1.49–3.21)	7 (left)	1.36 (0.25–2.47)	42.1	No
Tb.N	16	0.0010	3.30 (1.98–4.61)	6 (left)	1.94 (0.33–3.54)	41.2	No
Tb.Sp	15	0.0342	−2.92 (−4.01 to −1.83)	6 (right)	−1.99 (−3.18 to −0.80)	32.1	No
OCN	12	0.0512	2.11 (1.21–3.01)	5 (left)	1.33 (0.37–2.29)	37	No

Analyses used the same one-effect-per-study representative-dose dataset as the primary analyses. Trim-and-fill analyses were performed using the L0 estimator. Imputed studies represent hypothetical observations generated by the trim-and-fill procedure. For Tb. Sp, negative SMD, values indicate reduced trabecular separation with naringin; CI, confidence interval.

### 3.9 Certainty of evidence

Certainty was rated as low for CTX-1 and very low for all other outcomes (BMD, BV/TV, Tb.Th, Tb.N, Tb. Sp, OCN, P1NP, TRAP, and ALP). Outcome-level effect estimates, prediction intervals, domain judgements, and downgrading rationales are summarised in Table 10. Overall, no outcome reached moderate or high certainty because limitations related to study design, heterogeneity, indirectness, and reproducibility remained substantial.

**TABLE 10:** Certainty of evidence assessment using an adapted GRADE framework for preclinical animal studies.

Outcome	k	SMD 95% CI	Prediction interval	Risk of bias	Inconsistency	Imprecision	Indirectness	Publication bias	Overall certainty
BMD	22	2.30 (1.88–2.72)	0.72 to 3.87	Serious limitations	Not serious	Not serious	Serious	Serious concern (egger p = 0.0277)	Very low
BV/TV	17	3.36 (2.17–4.55)	−0.63 to 7.36	Serious limitations	Serious	Serious	Serious	Serious concern (egger p = 0.0077)	Very low
Tb.Th	17	2.35 (1.49–3.21)	−0.90 to 5.60	Serious limitations	Serious	Serious	Serious	Serious concern (egger p = 0.0282)	Very low
Tb.N	16	3.30 (1.98–4.61)	−1.37 to 7.96	Serious limitations	Serious	Serious	Serious	Serious concern (egger p = 0.0010)	Very low
Tb.Sp	15	−2.92 (−4.01 to −1.83)	−6.76 to 0.92	Serious limitations	Serious	Serious	Serious	Serious concern (egger p = 0.0342)	Very low
OCN	12	2.11 (1.21–3.01)	−0.77 to 4.99	Serious limitations	Serious	Not serious	Serious	Serious concern (egger p = 0.0512)	Very low
P1NP	7	2.32 (0.82–3.83)	−1.42 to 6.07	Serious limitations	Serious	Not serious	Serious	Not assessed (k < 10)	Very low
CTX-1	9	−2.38 (−2.97 to −1.79)	−3.00 to −1.76	Serious limitations	Not serious	Not serious	Serious	Not assessed (k < 10)	Low
TRAP	5	−3.67 (−6.81 to −0.53)	−10.44 to 3.10	Serious limitations	Serious	Serious	Serious	Not assessed (k < 10)	Very low
ALP	4	3.24 (−1.80–8.28)	Not estimated (k < 5)	Serious limitations	Serious	Serious	Serious	Not assessed (k < 10)	Very low

Certainty was initially rated as high and downgraded by one level for serious concerns and by two levels for very serious concerns. Risk of bias, inconsistency, imprecision, indirectness, and potential publication bias were assessed at the outcome level. Formal assessment of funnel-plot asymmetry was not performed for outcomes with fewer than 10 contributing studies.

All outcomes were downgraded for risk of bias because of unclear reporting in key SYRCLE domains and for indirectness because the evidence was limited to rodent models and surrogate outcomes without established correspondence to human exposure or clinical endpoints. BMD and CTX-1 were not downgraded for inconsistency because their primary-analysis prediction intervals excluded the null and their effect directions were comparatively stable. In contrast, BV/TV, Tb.Th, Tb.N, and Tb. Sp were downgraded for substantial unexplained heterogeneity and prediction intervals crossing the null, and additionally for potential small-study effects. OCN and P1NP also showed substantial heterogeneity despite generally consistent effect directions.

TRAP was further downgraded for inconsistency and imprecision because of the small evidence base, wide confidence and prediction intervals, and leave-one-out instability. ALP was downgraded for serious inconsistency and imprecision because only four studies contributed, heterogeneity was extreme, and the confidence interval included materially different effects in both directions. No outcome was upgraded because the large SMDs could not be separated from potential bias and no robust dose–response gradient was demonstrated.

## 4 Discussion

### 4.1 Overall effects and interpretation of evidence

This meta-analysis found favourable pooled effects of naringin on bone quantity, trabecular microarchitecture, and several bone turnover markers across animal models of osteoporosis. However, prediction intervals provided a more conservative assessment of reproducibility. Only BMD and CTX-1 had prediction intervals excluding the null, whereas those for BV/TV, Tb.Th, Tb.N, Tb. Sp, OCN, P1NP, and TRAP crossed the null; several model-specific subgroup estimates were also imprecise. Thus, the findings indicate a favourable overall preclinical signal, with comparatively stronger and more reproducible evidence for BMD and CTX‐1, while effects for several other outcomes may vary across experimental settings. This interpretation is consistent with the adapted GRADE framework, which rated most CTX‐1 as low certainty and all other outcomes as very low certainty due to limitations in methodological reporting, heterogeneity, indirectness of animal evidence, and imprecision.

Importantly, the evidence was not equally consistent across osteoporosis models. For Tb.Th, the significant interaction by model type and the small, non-significant estimate in the “other models” suggest potential variation in effects across model types. Across several structural outcomes, studies using diabetic, metabolic, or glucocorticoid-induced models tended to report smaller or near-null estimates; for example, Rodríguez et al. (2024) contributed near-null estimates for BV/TV, Tb.N, and Tb. Sp. In contrast, the larger pooled effects were derived predominantly from OVX and disuse models. Because the non-OVX subgroups contained relatively few and heterogeneous studies, this pattern cannot establish definitive effect modification. Nevertheless, it indicates that the current evidence is strongest for oestrogen-deficiency and disuse-related bone loss and remains substantially less certain for diabetic, metabolic, and glucocorticoid-induced osteoporosis.

Dose–response associations varied across modelling approaches, while the three-dimensional dose–duration plots were descriptive rather than inferential; their translational implications are discussed in Section 4.2. Confidence in the magnitude and generalisability of the pooled effects was further limited by unclear risk-of-bias judgements, potential small-study effects and selective reporting, substantial between-study heterogeneity, and inconsistent characterisation of naringin preparations.

### 4.2 Intervention characteristics, dose, and translational relevance

In addition to study-design limitations, incomplete characterisation of naringin materials represents a challenge to reproducibility. Although this review focused on isolated or purified naringin rather than botanical extracts, the source, supplier, purity, and analytical quality-control procedures were inconsistently reported across cohorts, limiting confidence that chemically comparable preparations were evaluated. Future studies should report metabolite authentication, purity assessment, and quality-control procedures transparently.

Reported naringin doses varied nearly 400-fold, from 4 to 1,500 mg/kg/day, precluding identification of a single translatable dose. Animal doses cannot be directly extrapolated to human therapeutic doses using body-weight conversion or simple human-equivalent dose (HED) calculations because absorption, metabolism, bioavailability, tissue distribution, and pharmacodynamic responses differ across species. Using body surface area conversion (rat Km = 6; human Km = 37), 100 mg/kg in rats corresponds to approximately 970 mg/day for a 60-kg adult. Nguyen et al. reported that 1,050 mg/day of naringin administered to healthy volunteers for 6 days was well tolerated (Nguyen et al., 2015). However, this short-term study primarily assessed safety and pharmacokinetic interactions rather than efficacy in osteoporosis. Moreover, animal doses of 300–1,500 mg/kg correspond to approximately 2.9–14.6 g/day for a 60-kg adult, exceeding available human pharmacokinetic and safety evidence. Such doses should therefore be regarded as experimental pharmacological exposures rather than clinically applicable doses.

HED conversion also fails to capture interspecies differences in metabolism and tissue exposure. After oral administration, naringin undergoes extensive metabolism, with naringenin and its conjugates representing major circulating forms (Chen et al., 2019). In healthy volunteers receiving 135 mg naringenin, peak plasma concentrations occurred after approximately 3.5 h, while cumulative urinary recovery was approximately 5.8%, indicating extensive metabolic transformation and limited systemic availability (Kanaze et al., 2007). Comparative pharmacokinetic analyses further demonstrate substantial species differences in naringin/naringenin exposure, limiting the validity of dose normalisation for predicting human exposure (Zhang et al., 2020). In addition, first-pass metabolism and gut microbiota-mediated conversion of naringin to naringenin vary between individuals and species (Nair and Jacob, 2016), further complicating cross-species extrapolation.

The present analyses did not identify a stable optimal dosing window. Significant dose coefficients differed between the raw-dose and log10-dose models, while influence diagnostics and high-dose exclusions indicated sensitivity to sparse observations at the upper end of the dose range. Only BMD had sufficient study clusters for adjustment by species; although its dose coefficient remained significant, the model included only 5 mouse studies, retained significant residual heterogeneity, and could not be adjusted for osteoporosis model type. The three-dimensional dose–duration plots were descriptive and were not used to infer dose–response relationships. Furthermore, most studies were not designed as formal dose-ranging experiments, provided insufficient information to determine a minimum effective dose, and inconsistently reported positive controls.

Consequently, the available evidence does not establish a dose–response relationship, an optimal dose, or a starting dose for clinical translation. Future studies should use dedicated within-study dose-ranging designs integrated with pharmacokinetic and pharmacodynamic assessments—including tissue exposure, metabolite profiles, and long-term safety—before naringin is considered for clinical development in osteoporosis.

### 4.3 Potential mechanisms of Naringin’s skeletal effects

Beyond the quantitative findings, experimental studies suggest that naringin may influence both osteoblast-mediated bone formation and osteoclast-mediated bone resorption. In osteoporosis, ageing, oestrogen deficiency, oxidative stress, inflammation, glucocorticoid exposure, and impaired Wnt/β-catenin signalling disrupt bone remodelling, leading to bone loss, deterioration of trabecular microarchitecture, and increased fracture susceptibility (Figure 14). Naringin has been proposed to counter this imbalance through coordinated osteogenic and anti-resorptive mechanisms (Yan et al., 2026).

**FIGURE 14:**
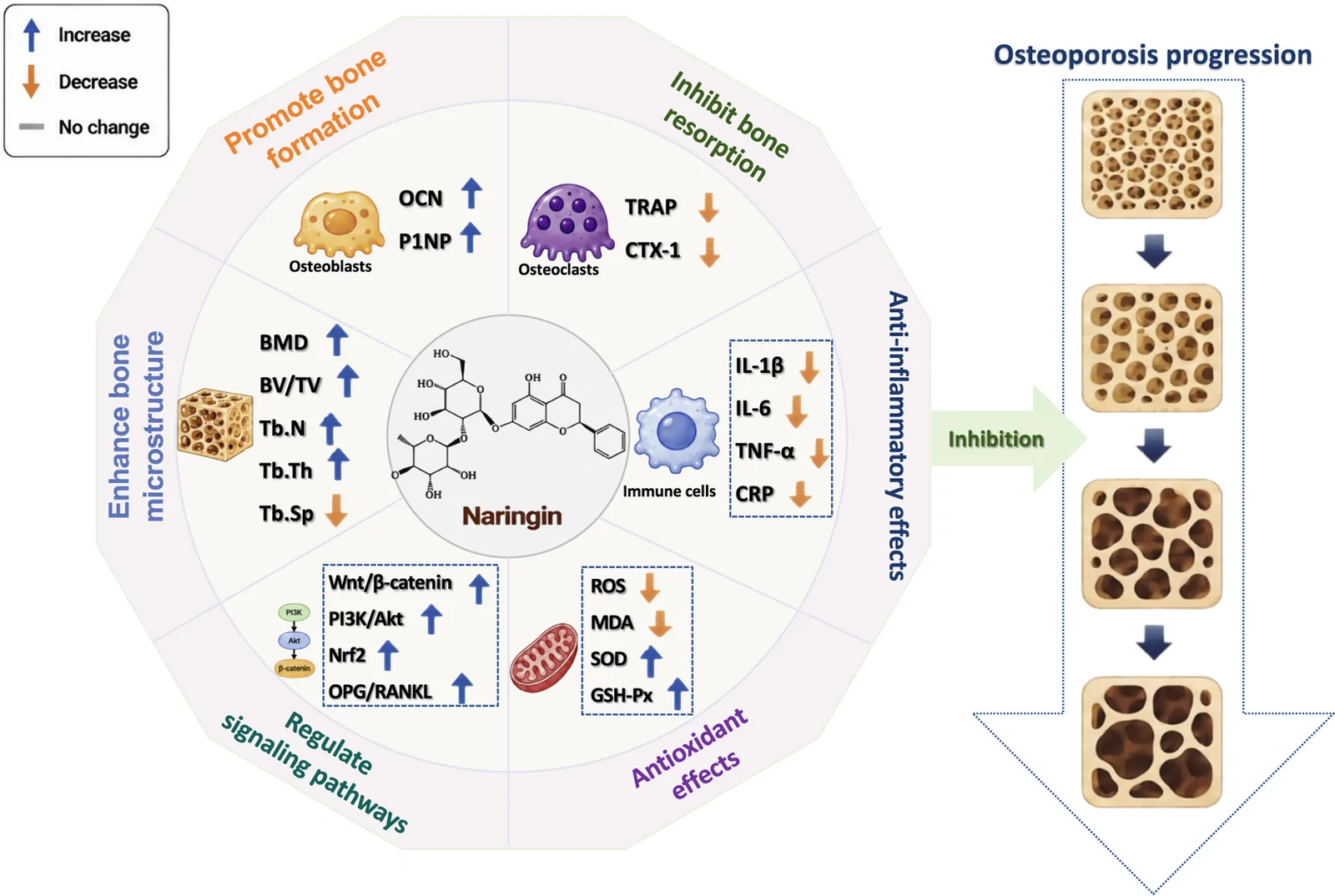
Putative mechanisms underlying the skeletal effects of naringin. Dashed-line boxes indicate potential biological mechanisms proposed by previous experimental studies but not directly evaluated in this meta-analysis. Therefore, these mechanisms should be interpreted as potential explanations for the observed skeletal effects rather than causal mechanisms established by the present study.

#### 4.3.1 Promoting osteoblast-mediated bone formation

Mechanistic studies indicate that naringin may promote osteoblast differentiation by stabilising β-catenin through GSK-3β inhibition and upregulating RUNX2 and downstream matrix proteins (Cui et al., 2025). The PI3K/Akt/mTOR and JAK2/STAT3 pathways have also been implicated in its osteogenic effects (Ge and Zhou, 2021; Wang et al., 2022). In osteoblast-lineage cells exposed to oxidative stress, naringin increased GSK-3β phosphorylation and β-catenin accumulation through concurrent activation of the Wnt/β-catenin and PI3K/Akt pathways, thereby enhancing osteogenic differentiation (Wang et al., 2024).

The favourable pooled estimates for BMD, trabecular microarchitecture, OCN, and P1NP are compatible with these proposed formation-promoting mechanisms. However, prediction intervals crossed the null for all corresponding outcomes except BMD. These findings therefore provide only indirect biological support, as signalling-pathway activity was not assessed and causality cannot be established by the present meta-analysis.

#### 4.3.2 Inhibiting osteoclast-mediated bone resorption

Naringin has also been reported to regulate the RANKL/OPG axis by increasing OPG expression and reducing the RANKL/OPG ratio, while suppressing osteoclast differentiation through NF-κB inhibition and downregulation of cathepsin K and matrix metalloproteinase-9 (Yu et al., 2020). A subsequent review further described inhibition of RANKL-induced NF-κB signalling through reduced IκBα degradation and dose-dependent downregulation of osteoclast-related genes, including cathepsin K and TRAP (Gong et al., 2024).

The meta-analytic findings were partly compatible with these anti-resorptive mechanisms. CTX-1 showed a favourable pooled reduction with moderate heterogeneity and a prediction interval excluding the null. Although TRAP also showed a favourable pooled mean reduction, its prediction interval crossed the null and its significance was not retained in three of five leave-one-out analyses. Evidence for ALP was inconclusive because only four studies contributed, heterogeneity was extreme, and the confidence interval encompassed materially different effects in both directions. Moreover, serum ALP is a non-specific marker that may also originate from hepatic or intestinal sources (Eastell and Szulc, 2017); its positive point estimate should therefore not be interpreted as evidence of enhanced osteoblast activity.

Overall, the observed changes in formation- and resorption-related outcomes are compatible with naringin acting on both components of bone remodelling. Nevertheless, the present analysis cannot establish causal relationships or confirm the involvement of specific signalling pathways.

### 4.4 Limitations and future research directions

Several limitations constrain the interpretation and clinical translation of these findings. Funnel-plot asymmetry and the 8.7%–42.1% attenuation of effect estimates after trim-and-fill suggest that some pooled magnitudes may be overestimated. However, these findings do not establish publication bias or predict reproducibility because both methods are sensitive to substantial heterogeneity and the mathematical association between SMD and its standard error. Risk-of-bias assessments were also dominated by unclear judgements. This should not be interpreted as evidence of low risk, as inadequate reporting prevented reliable assessment of randomisation, allocation concealment, housing, blinding, and selective reporting.

Considerable heterogeneity persisted for most secondary outcomes despite subgroup and sensitivity analyses. This probably reflects multiple sources of biological and experimental variation—including animal species and strains, osteoporosis models, dose ranges, treatment durations, and measurement methods—rather than a single correctable factor. Inconsistent reporting of naringin source, supplier, purity, and analytical quality control further limits the comparability of nominal doses and may contribute to unexplained heterogeneity. Moreover, the evidence was derived exclusively from rodent models and surrogate skeletal or biochemical outcomes, limiting direct extrapolation to human osteoporosis.

Several analytical decisions, including dose-arm selection and post-hoc sensitivity analyses, were exploratory. Although pooled mean effects were generally stable across alternative analyses, dose-related findings varied across specifications and should therefore be considered hypothesis-generating rather than confirmatory. publications may also have excluded eligible studies indexed in other regional databases.

Future studies should prioritise adequately powered, prospectively registered dose-ranging experiments using chemically characterised naringin, with predefined sample-size calculations, randomisation, blinded outcome assessment, and appropriate controls. Independent replication is particularly needed in glucocorticoid‐induced, diabetic, and metabolic models. Pharmacokinetic, pharmacodynamic, tissue-exposure, metabolite-profile, and safety assessments should also be incorporated to improve reproducibility and translational relevance.

## 5 Conclusion

This systematic review and This systematic review and meta-analysis identified favourable pooled effects of naringin on bone mineral density, trabecular microarchitecture, and several bone turnover markers in animal models of osteoporosis. The overall direction of effects was generally preserved across sensitivity analyses, with comparatively robust evidence for BMD and CTX‐1. These findings support a broad skeletal-protective potential of naringin in preclinical osteoporosis models and are biologically compatible with effects on both bone formation and resorption. However, heterogeneity, prediction intervals crossing the null for several outcomes, and low certainty of evidence indicate that the magnitude and reproducibility of these effects remain uncertain across experimental settings. Further well-designed preclinical studies incorporating standardised naringin characterisation, dose-ranging approaches, pharmacokinetic assessment, and safety evaluation are warranted to strengthen the evidence base and inform future translational research.

## Data Availability

The original contributions presented in the study are included in the article/Supplementary Material, further inquiries can be directed to the corresponding authors.

## Appendix A: Records excluded following full-text assessment and reasons for exclusion

**Table:**

First author, year	Report title	Journal/DOI	Primary reason for exclusion
Shangguan et al., 2022	Naringin exerts anti-osteoporosis effects by promoting H-type angiogenesis through HIF-1α/VEGF signalling	Chinese journal of osteoporosis, 28, 1755–1759 DOI: 10.3969/j.issn.1006-7108.2022.12.009	Duplicate record identified during full-text review. The English-indexed and Chinese records referred to the same publication and were counted only once
Guo et al., 2011	Double directional adjusting estrogenic effect of naringin from rhizoma drynariae (gusuibu)	Journal of ethnopharmacology, 138 (2), 451–457 DOI: 10.1016/j.jep.2011.09.034	No eligible osteoporosis or bone-loss animal model. The study evaluated estrogenic activity using cell-based assays and a uterotrophic mouse experiment rather than treatment of an osteoporosis model
Song et al., 2017	Naringin promotes fracture healing through stimulation of angiogenesis by regulating the VEGF/VEGFR-2 signaling pathway in osteoporotic rats	Chemico-biological interactions, 261, 11–17 DOI: 10.1016/j.cbi.2016.10.020	Ineligible condition and outcomes. The study evaluated fracture healing and callus angiogenesis in osteoporotic rats rather than the treatment of osteoporosis or generalized bone loss
