# Supplementary material for: Preclinical effects of naringin on bone outcomes in animal models of osteoporosis: a systematic review and meta-analysis
Source: Front Pharmacol. 2026 Sep 18;17:1945663. doi: 10.3389/fphar.2026.1945663 (PMC13630924; doi:10.3389/fphar.2026.1945663)
Supplement: Supplementary file 1 [file DataSheet1.docx]

# Supplementary Material

# Contents

Supplementary 1. Complete search strategies across the five databases

Supplementary 2. Study-level meta-regression of naringin dose on effect size

Supplementary 3. Exploratory subgroup analyses by osteoporosis model type

Supplementary 4. Post-hoc sensitivity analyses excluding outlier studies

Supplementary 5. PRISMA 2020 checklist

**Supplementary 1**

**Table S1.** **Complete search strategies across the five databases, including search term groups and date ranges**

| **No.** | **Database** | **Language / Type** | **Date Range** |
| --- | --- | --- | --- |
| ① | PubMed | English / Biomedical | January 2010 – May 2026 |
| ② | Web of Science | English / Multidisciplinary | January 2010 – May 2026 |
| ③ | Embase | English / Pharmacology-focused | January 2010 – May 2026 |
| ④ | CNKI (China National Knowledge Infrastructure) | Chinese | January 2010 – May 2026 |
| ⑤ | Wanfang Data | Chinese | January 2010 – May 2026 |

**Table S2. Term Grouping**

| **Group** | **Concept** | **Search Terms (MeSH + Free Text)** |
| --- | --- | --- |
| **Group A** | **Intervention** | MeSH: "Naringin"[MeSH Terms]  Free text: naringin, naringenin-7-rhamnoglucoside,  naringin-7-O-neohesperidoside  Exclude: naringenin (CAS 480-41-1; a structurally distinct aglycone);  isonaringin (a synonym for narirutin, NOT naringin — verify at  screening stage) |
| **Group B** | **Disease / Condition** | MeSH: "Osteoporosis"[MeSH Terms], "Bone Diseases, Metabolic"[MeSH Terms],  "Bone Resorption"[MeSH Terms]  Free text: osteoporosis, osteopenia, bone loss, bone density,  bone mineral density, BMD, bone metabolism,  bone remodeling, postmenopausal bone loss |
| **Group C** | **Animal Models** | MeSH: "Disease Models, Animal"[MeSH Terms], "Ovariectomy"[MeSH Terms],  "Rats"[MeSH Terms], "Mice"[MeSH Terms]  Free text: animal model, rat, rats, mice, mouse, murine,  ovariectomy, ovariectomized, OVX,  glucocorticoid-induced, corticosteroid-induced,  dexamethasone-induced, hindlimb suspension,  sciatic neurectomy, disuse, streptozotocin, STZ,  diabetic bone loss, high-fructose, metabolic syndrome,  retinoic acid-induced |
| ** Naringin (CAS 10236-47-2) and naringenin (CAS 480-41-1) are chemically distinct compounds. Searches were restricted to naringin; naringenin-related records were excluded at both the search construction and title/abstract screening stages. isonaringin is a synonym for narirutin (naringenin-7-O-rutinoside) and must not be treated as a synonym for naringin; records identified via isonaringin were re-verified at the full-text stage.* | | |

**Table S3. Complete Search Strings by Database**

**PubMed Search String**

("Naringin"[MeSH Terms] OR "naringin"[All Fields] OR

"naringenin-7-rhamnoglucoside"[All Fields] OR "naringin-7-O-neohesperidoside"[All Fields])

AND

("Osteoporosis"[MeSH Terms] OR "Bone Diseases, Metabolic"[MeSH Terms] OR

"Bone Resorption"[MeSH Terms] OR "osteoporosis"[All Fields] OR

"osteopenia"[All Fields] OR "bone loss"[All Fields] OR

"bone mineral density"[All Fields] OR "BMD"[All Fields] OR

"bone metabolism"[All Fields] OR "bone remodeling"[All Fields] OR

"trabecular bone"[All Fields] OR "cortical bone"[All Fields])

AND

("Disease Models, Animal"[MeSH Terms] OR "Ovariectomy"[MeSH Terms] OR

"Rats"[MeSH Terms] OR "Mice"[MeSH Terms] OR

"animal model"[All Fields] OR "rat"[All Fields] OR

"mice"[All Fields] OR "mouse"[All Fields] OR

"ovariectomy"[All Fields] OR "ovariectomized"[All Fields] OR "OVX"[All Fields] OR

"glucocorticoid-induced"[All Fields] OR "dexamethasone-induced"[All Fields] OR

"corticosteroid-induced"[All Fields] OR "retinoic acid"[All Fields] OR

"hindlimb suspension"[All Fields] OR "sciatic neurectomy"[All Fields] OR

"disuse"[All Fields] OR "streptozotocin"[All Fields] OR

"diabetic bone loss"[All Fields] OR "high-fructose"[All Fields])

AND ("2010/01/01"[Date - Publication] : "3000/12/31"[Date - Publication])

**Web of Science Search String**

TS=("naringin" OR "naringenin-7-rhamnoglucoside" OR "naringin-7-O-neohesperidoside")

AND TS=("osteoporosis" OR "osteopenia" OR "bone loss" OR "bone mineral density"

OR "BMD" OR "bone metabolism" OR "bone remodeling" OR "bone resorption"

OR "trabecular bone" OR "cortical bone")

AND TS=("animal model" OR "rat" OR "rats" OR "mice" OR "mouse" OR "murine"

OR "ovariectomy" OR "ovariectomized" OR "OVX"

OR "glucocorticoid-induced" OR "dexamethasone-induced"

OR "corticosteroid-induced" OR "retinoic acid"

OR "hindlimb suspension" OR "sciatic neurectomy" OR "disuse"

OR "streptozotocin" OR "diabetic bone loss" OR "high-fructose")

AND PY=(2010-2026)

**Embase Search String**

('naringin'/exp OR 'naringin' OR 'naringenin-7-rhamnoglucoside'

OR 'naringin-7-O-neohesperidoside')

AND

('osteoporosis'/exp OR 'osteoporosis' OR 'osteopenia' OR 'bone loss'

OR 'bone mineral density' OR 'bmd' OR 'bone metabolism' OR 'bone remodeling'

OR 'trabecular bone' OR 'cortical bone')

AND

('animal model'/exp OR 'ovariectomy'/exp OR 'rat'/exp OR 'mouse'/exp

OR 'ovariectomy' OR 'ovariectomized' OR 'ovx'

OR 'glucocorticoid induced' OR 'dexamethasone induced'

OR 'corticosteroid induced' OR 'retinoic acid'

OR 'hindlimb suspension' OR 'sciatic neurectomy' OR 'disuse'

OR 'streptozotocin' OR 'diabetic bone loss' OR 'high fructose')

AND [2010-2026]/py

**CNKI (China National Knowledge Infrastructure) — Advanced Search Settings**

Search field: Topic

Term 1: 柚皮苷 [naringin] (fuzzy match)

AND Term 2: 骨质疏松 [osteoporosis]

AND Term 3: 大鼠 OR 小鼠 OR 动物 OR 去卵巢 OR OVX OR 糖皮质激素 OR 废用性

OR 糖尿病 OR STZ OR 链脲佐菌素 OR 后肢悬吊 OR 高果糖

[rat OR mouse OR animal OR ovariectomy OR OVX OR glucocorticoid

OR disuse OR diabetes OR STZ OR streptozotocin

OR hindlimb suspension OR high-fructose]

Publication date: 1 January 2010 – May 2026

**Wanfang Data — Advanced Search Settings**

Search field: Topic

Search string: 柚皮苷 AND 骨质疏松 AND

（大鼠 OR 小鼠 OR 动物模型 OR 去卵巢 OR 糖皮质激素 OR 废用性

OR 糖尿病 OR 链脲佐菌素 OR 后肢悬吊 OR 高果糖）

[naringin AND osteoporosis AND

(rat OR mouse OR animal model OR ovariectomy OR glucocorticoid OR disuse

OR diabetes OR streptozotocin OR hindlimb suspension OR high-fructose)]

Publication date: January 2010 – May 2026

**Supplementary 2**

| **Outcome** | **k** | **Slope** | **95% CI** | **p** | **I²(resid)** | **R²** |
| --- | --- | --- | --- | --- | --- | --- |
| BMD | 22 | 0.005 | 0.0004, 0.0096 | 0.034 | 49.50% | 28.60% |
| BV/TV | 17 | 0.005 | −0.0061, 0.0161 | 0.377 | 87.20% | 0% |
| Tb.Th | 17 | 0.0102 | 0.0027, 0.0176 | 0.007 | 77.50% | 33.50% |
| Tb.N | 16 | 0.0084 | −0.0046, 0.0214 | 0.206 | 90.10% | 3.80% |
| Tb.Sp | 15 | −0.0112 | −0.0212, −0.0012 | 0.028 | 82.00% | 25.40% |
| OCN | 12 | 0.0009 | −0.0080, 0.0098 | 0.839 | 81.50% | 0% |
| *^*^ baseline model for the highest-dose-arm sensitivity comparison in Section 3.8* | | | | | | |

**Table S4. Representative-dose, one-effect-per-study meta-regression of naringin dose (mg/kg/day) on effect size across 6 outcomes**

**Supplementary 3**

## （A）

## （B）

**Figure S1.** Post-hoc sensitivity analyses excluding studies with |SMD| > 8 identified upon visual inspection of forest plots. (A) BV/TV; (B) Tb.N .

**Supplementary 4**

## （A）

## （B）

## （C）

## （D）

**Figure S2.** Exploratory subgroup analyses by osteoporosis model type for secondary structural outcomes and OCN. (A) Tb.Th; (B) Tb.N; (C) Tb.Sp; (D) OCN.

**Supplementary 5**

**Table S5. PRISMA 2020 checklist**

| Section and Topic | Item # | Checklist item | Location where item is reported |
| --- | --- | --- | --- |
| TITLE | | |  |
| Title | 1 | Identify the report as a systematic review. | Title page. |
| ABSTRACT | | |  |
| Abstract | 2 | See the PRISMA 2020 for Abstracts checklist. | Abstract (structured Background/Methods/Results/Conclusion). |
| INTRODUCTION | | |  |
| Rationale | 3 | Describe the rationale for the review in the context of existing knowledge. | Section 1 (Introduction), paragraphs 2–5. |
| Objectives | 4 | Provide an explicit statement of the objective(s) or question(s) the review addresses. | Section 1 (Introduction), final paragraph. |
| METHODS | | |  |
| Eligibility criteria | 5 | Specify the inclusion and exclusion criteria for the review and how studies were grouped for the syntheses. | Section 2.3 (Inclusion and Exclusion Criteria). |
| Information sources | 6 | Specify all databases, registers, websites, organisations, reference lists and other sources searched or consulted to identify studies. Specify the date when each source was last searched or consulted. | Section 2.2 (Search Strategy), paragraph 1. The publication-date coverage window (January 2010–May 2026) is reported. |
| Search strategy | 7 | Present the full search strategies for all databases, registers and websites, including any filters and limits used. | Section 2.2 (Search Strategy); full search strings for all five databases are provided in the Supplementary Materials. |
| Selection process | 8 | Specify the methods used to decide whether a study met the inclusion criteria of the review, including how many reviewers screened each record and each report retrieved, whether they worked independently, and if applicable, details of automation tools used in the process. | Section 2.4 (Study Selection). |
| Data collection process | 9 | Specify the methods used to collect data from reports, including how many reviewers collected data from each report, whether they worked independently, any processes for obtaining or confirming data from study investigators, and if applicable, details of automation tools used in the process. | Section 2.6 (Data Extraction), paragraph 1. Two reviewers independently digitized values reported only in figures. |
| Data items | 10a | List and define all outcomes for which data were sought. Specify whether all results that were compatible with each outcome domain in each study were sought (e.g. for all measures, time points, analyses), and if not, the methods used to decide which results to collect. | Section 2.5 (Outcome Measures). |
|  | 10b | List and define all other variables for which data were sought (e.g. participant and intervention characteristics, funding sources). Describe any assumptions made about any missing or unclear information. | Section 2.6 (Data Extraction), paragraph 1 (animal and intervention variables). |
| Study risk of bias assessment | 11 | Specify the methods used to assess risk of bias in the included studies, including details of the tool(s) used, how many reviewers assessed each study and whether they worked independently, and if applicable, details of automation tools used in the process. | Section 2.7 (Risk of Bias Assessment). |
| Effect measures | 12 | Specify for each outcome the effect measure(s) (e.g. risk ratio, mean difference) used in the synthesis or presentation of results. | Section 2.8 (Statistical Analysis), paragraph 1. |
| Synthesis methods | 13a | Describe the processes used to decide which studies were eligible for each synthesis (e.g. tabulating the study intervention characteristics and comparing against the planned groups for each synthesis (item #5)). | Sections 2.3, 2.5, 2.6, and 2.8. |
|  | 13b | Describe any methods required to prepare the data for presentation or synthesis, such as handling of missing summary statistics, or data conversions. | Section 2.6 (Data Extraction). |
|  | 13c | Describe any methods used to tabulate or visually display results of individual studies and syntheses. | Section 2.8 (Statistical Analysis), particularly paragraphs 1, 3, and 4; Tables 1–9 and Figures 3–13 in the main text; Supplementary Figures S1–S2. |
|  | 13d | Describe any methods used to synthesize results and provide a rationale for the choice(s). If meta-analysis was performed, describe the model(s), method(s) to identify the presence and extent of statistical heterogeneity, and software package(s) used. | Section 2.8 (Statistical Analysis), paragraphs 1–3 and 6–7. |
|  | 13e | Describe any methods used to explore possible causes of heterogeneity among study results (e.g. subgroup analysis, meta-regression). | Section 2.8 (Statistical Analysis), paragraphs 2–3 and 6–7. |
|  | 13f | Describe any sensitivity analyses conducted to assess robustness of the synthesized results. | Section 2.8 (Statistical Analysis), paragraphs 4–7. |
| Reporting bias assessment | 14 | Describe any methods used to assess risk of bias due to missing results in a synthesis (arising from reporting biases). | Section 2.8 (Statistical Analysis), paragraph 4 (funnel plots, Egger’s test, trim-and-fill). |
| Certainty assessment | 15 | Describe any methods used to assess certainty (or confidence) in the body of evidence for an outcome. | Section 2.9 (Certainty of Evidence). |
| RESULTS | | |  |
| Study selection | 16a | Describe the results of the search and selection process, from the number of records identified in the search to the number of studies included in the review, ideally using a flow diagram. | Section 3.1 (Literature Selection); Figure 1 (PRISMA 2020 flow diagram). |
|  | 16b | Cite studies that might appear to meet the inclusion criteria, but which were excluded, and explain why they were excluded. | Section 3.1 (Literature Selection), final sentence; Appendix A. |
| Study characteristics | 17 | Cite each included study and present its characteristics. | Section 3.2 (Characteristics of Included Studies); Tables 1–2. |
| Risk of bias in studies | 18 | Present assessments of risk of bias for each included study. | Section 3.3 (Risk of Bias); Figure 2. |
| Results of individual studies | 19 | For all outcomes, present, for each study: (a) summary statistics for each group (where appropriate) and (b) an effect estimate and its precision (e.g. confidence/credible interval), ideally using structured tables or plots. | Sections 3.4–3.5; Figures 3, 5, and 7–9. Effect estimates and 95% CIs are shown, but group-level summary statistics (e.g., n, mean, and SD) are not presented for every study and outcome; this item is only partially reported. |
| Results of syntheses | 20a | For each synthesis, briefly summarise the characteristics and risk of bias among contributing studies. | Sections 3.2–3.5 and Section 3.3 (Risk of Bias). The characteristics and risk of bias of studies contributing to each individual synthesis are only partially summarised. |
|  | 20b | Present results of all statistical syntheses conducted. If meta-analysis was done, present for each the summary estimate and its precision (e.g. confidence/credible interval) and measures of statistical heterogeneity. If comparing groups, describe the direction of the effect. | Sections 3.4–3.8; Tables 4–8; Figures 3–13; Supplementary Figures S1–S2. |
|  | 20c | Present results of all investigations of possible causes of heterogeneity among study results. | Sections 3.4–3.6; Figures 4, 6, and 10; Table 5; Supplementary Figure S1. |
|  | 20d | Present results of all sensitivity analyses conducted to assess the robustness of the synthesized results. | Section 3.8; Tables 4 and 6–8; Figure 12; Supplementary Figure S2. |
| Reporting biases | 21 | Present assessments of risk of bias due to missing results (arising from reporting biases) for each synthesis assessed. | Section 3.8 (Sensitivity Analyses and Assessment of Small-study Effects); Table 7, Figure 13. |
| Certainty of evidence | 22 | Present assessments of certainty (or confidence) in the body of evidence for each outcome assessed. | Section 3.9 (Certainty of Evidence); Table 9. |
| DISCUSSION | | |  |
| Discussion | 23a | Provide a general interpretation of the results in the context of other evidence. | Section 4.1 (Overall Effects and Interpretation of Evidence). |
|  | 23b | Discuss any limitations of the evidence included in the review. | Section 4.4 (Limitations and Future Research Directions), paragraphs 1–2. |
|  | 23c | Discuss any limitations of the review processes used. | Section 4.4 (Limitations and Future Research Directions), paragraph 3. |
|  | 23d | Discuss implications of the results for practice, policy, and future research. | Section 4.4 (Limitations and Future Research Directions), final paragraph. |
| OTHER INFORMATION | | |  |
| Registration and protocol | 24a | Provide registration information for the review, including register name and registration number, or state that the review was not registered. | Section 2.1 (Protocol Registration); also Section 1 (Introduction), final paragraph, and Data Availability Statement. PROSPERO CRD420261419299. |
|  | 24b | Indicate where the review protocol can be accessed, or state that a protocol was not prepared. | Section 2.1 provides the PROSPERO registration number (CRD420261419299). |
|  | 24c | Describe and explain any amendments to information provided at registration or in the protocol. | Section 2.5 (Outcome Measures): P1NP, TRAP, and ALP were added during the review. |
| Support | 25 | Describe sources of financial or non-financial support for the review, and the role of the funders or sponsors in the review. | Funding section. |
| Competing interests | 26 | Declare any competing interests of review authors. | Conflict of Interest section. |
| Availability of data, code and other materials | 27 | Report which of the following are publicly available and where they can be found: template data collection forms; data extracted from included studies; data used for all analyses; analytic code; any other materials used in the review. | Data Availability Statement. The manuscript states that relevant materials are included in the article and Supplementary Material and that further inquiries may be directed to the corresponding authors. |
